# Veterinary education and experience shape beliefs about dog breeds Part 1: Pain sensitivity

**DOI:** 10.1038/s41598-023-40671-y

**Published:** 2023-08-24

**Authors:** Rachel M. P. Caddiell, Philip White, B. Duncan X. Lascelles, Kenneth Royal, Kimberly Ange-van Heugten, Margaret E. Gruen

**Affiliations:** 1 Comparative Behavioral Research, Department of Clinical Sciences, College of Veterinary Medicine, North Carolina State University, Raleigh, NC USA; 2 Department of Clinical Sciences, College of Veterinary Medicine, North Carolina State University, Raleigh, NC USA; 3 Translational Research in Pain, Department of Clinical Sciences, College of Veterinary Medicine, North Carolina State University, Raleigh, NC USA; 4 Department of Statistics, College of Physical and Mathematical Sciences, Brigham Young University, Provo, UT USA; 5 Comparative Pain Research and Education Center, College of Veterinary Medicine, North Carolina State University, Raleigh, NC USA; 6 Thurston Arthritis Centre, UNC School of Medicine, Chapel Hill, NC USA; 7 Department of Anesthesiology, Center for Translational Pain Research, Duke University, Durham, NC USA; 8 Department of Animal Science, College of Agriculture and Life Sciences, North Carolina State University, Raleigh, NC USA; 9 Environmental Medicine Consortium, North Carolina State University, Raleigh, NC USA

**Keywords:** Human behaviour, Health care

## Abstract

Over 95% of veterinarians report believing that dog breeds differ in pain sensitivity. Ratings made by veterinarians differ from those of the general public, suggesting these beliefs may be learned during veterinary training or clinical experiences. Therefore, the current study’s primary objective was to evaluate dog breed pain sensitivity ratings during veterinary training and compare these ratings to those of the general public and undergraduates in animal-health related fields. Using an online survey, members of the general public, undergraduates, veterinary students across all four years, and veterinary faculty and staff rated pain sensitivity of 10 different dog breeds, identified only by their pictures. Compared to the general public and undergraduates, veterinary students rated pain sensitivity across breeds of dog more similarly to veterinary faculty and staff. Further, when undergraduates had clinical experience, they also rated certain dog breeds in a similar way to the veterinary students and professionals. Our findings suggest that veterinary education and clinical experiences influence pain sensitivity ratings across dog breeds. Future research should identify how these pain sensitivity beliefs are communicated and whether these beliefs affect recognition and treatment of pain by veterinarians.

## Introduction

Pain sensitivity in dogs is believed to vary based on breed-specific stereotypes (i.e., commonly held beliefs). In 2020, Gruen et al.^1^ surveyed over 1000 members of the general public and 1000 veterinarians and found that across 28 dog breeds, different breeds were rated as having varying degrees of pain sensitivity. Breed-specific pain sensitivity ratings by the general public were primarily related to dog size (with smaller dogs being rated as having higher pain sensitivity) and presence of breed-specific legislation (i.e., laws that regulate and/or ban certain dog breeds). Among veterinarian respondents, breed-specific pain sensitivity ratings differed from the general public with veterinarians reporting distinct pain sensitivity ratings that were highly consistent with one another, even when accounting for time since graduation. This suggests substantial agreement in beliefs about dog breed pain sensitivity within the profession. Given that there was close agreement amongst veterinarians regarding perceptions about dog breed pain sensitivity, and that these perceptions differed from the general public, educational factors may play a role in shaping these perceptions. Additional support for this hypothesis is that veterinarians who recently graduated from veterinary school reported similar ratings to those who had graduated 25 years prior. To date, no research has been performed to understand what shapes veterinarians’ beliefs about dog breed pain sensitivity.

While not yet studied in veterinary medicine, there is substantial literature in human medicine regarding stereotypes in pain sensitivity ratings. In the United States health care system, disparities exist related to pain recognition, treatment, and management^2,3^. These care disparities have been attributed to systemic issues, such as access to healthcare^4,5^, as well as health care worker’s beliefs about pain sensitivity in others^5,6^. When assessing pain in other humans, there is a tendency to rely on stereotypes related to identities including race, ethnicity, gender, and socioeconomic status to justify beliefs about why some groups do not feel pain equally^7–11^. Previous work has identified that health care workers rate pain sensitivity differently in patients of different races, genders, and socioeconomic backgrounds^7,9,12,13^. In response to the evidence of healthcare worker bias, training programs have implemented cultural competency courses designed to increase awareness of healthcare disparities; however, there is little indication that these aspects of formal education are helpful in reducing bias^14–16^. Further, recent research has suggested that exposure to the “culture of medicine” during training programs may increase bias in medical students^17–19^.

To date, there has not been a comprehensive survey regarding pain education curricula across health care professions in the United States^20^. However, data from Canada and the United Kingdom suggest that veterinarians receive anywhere from two to five times more knowledge about pain compared to human medical physicians during their formal education^21,22^. This figure may be similar for veterinarians in the United States as pain management is recognized as a component of one of the nine clinical competencies expected of veterinary students upon graduation^23^. Still, veterinary professionals frequently report having inadequate knowledge of pain assessment as a significant barrier to treating pain^24–27^. Therefore, breed-specific pain stereotypes may be used by veterinarians to aid in pain assessment when clinical uncertainty exists. While further work will be needed to understand whether these beliefs have any impact on care, it is imperative to first understand if medical training plays a role in veterinarians acquiring and/or strengthening these breed-specific pain stereotypes, as is the case in human medicine.

The present study aimed to evaluate pain sensitivity ratings during veterinary medical training and compare these ratings to those of the general public, and undergraduates studying in animal-health related fields. These comparisons were designed to determine if differences exist across the years of veterinary training and if so, identify when during training changes in perceptions develop. Undergraduates with and without veterinary clinical experience were also included to ascertain the influence of veterinary experience outside of a veterinary school. We predicted that we would find differences between ratings from the general public and veterinary faculty and staff similar to previous findings^1^. This includes assessment of the relationship between pain sensitivity ratings and how warm or cool respondents felt about a breed, as assessed by feelings thermometers. Our previous work found a significant relationship between feelings thermometer ratings and pain sensitivity ratings^1^. We hypothesized that veterinary students would rate pain sensitivity progressively more similar to veterinary faculty and staff as they progressed in their training program. Additionally, we predicted that clinical experience would affect pain sensitivity ratings with students who had clinical experience reporting pain sensitivity ratings more similar to veterinary faculty and staff.

## Methods

We purposefully sampled from four distinct populations for this study: members of the general public, undergraduate students at North Carolina State University in majors pursued by individuals wanting a career with animals, veterinary students, and veterinary school faculty and staff. The study was categorized as exempt by the North Carolina State University’s Institutional Review Board (Protocol #22285) and was performed in accordance with all relevant guidelines and regulations. Prior to answering any survey questions, participants were presented with an introductory statement that included an explanation of the study and were asked to indicate informed consent for survey responses to be used in this research.

Members of the general public were recruited through Amazon Mechanical Turk (mTurk), a crowdsourcing marketplace, via Amazon.com. This platform is comparable to other methods of obtaining survey participants and prior research has indicated that mTurk samples offer socioeconomically and culturally diverse participants^28–30^. To ensure responses were legitimate, an attention check question was inserted following the third block of questions (described below). For the present study, a sample of 1020 mTurk participants were obtained to yield a margin-of-error within 3%. Each mTurk participant who fully completed the survey and passed the attention check received a nominal fee ($0.75) for their participation. The survey was open to respondents from October 25, 2020, to November 4, 2020.

Undergraduate students were recruited from North Carolina State University (NCSU) within the following majors: Animal Science, Zoology, and Biology. These majors were selected as they are likely to include students with knowledge of animals and an interest in animal-focused careers (including veterinary medicine). Veterinary students, and veterinary faculty and staff members (including veterinary interns and residents) were recruited from North Carolina State University, Auburn University, University of Georgia, Iowa State University, Louisiana State University, Oregon State University, Tufts University, and Virginia Tech University. All responses were collected anonymously. Students who completed the survey were invited to provide their email to one investigator (RMPC) to be entered in a raffle to win a $20 gift card. A total of 39 gift cards were awarded. No incentives were provided for faculty or staff members. The length of survey availability differed by university. The survey was open to NCSU respondents from November 13, 2020, to January 18, 2021. The survey was open to the remaining universities for an average of 3.5 weeks with the dates of access variable dependent on when their administration agreed to send out the survey to veterinary students, and veterinary faculty and staff members at their institution. Reminder emails were sent to encourage survey participation. The survey yielded a final sample of 361 undergraduates, 536 veterinary students, and 293 veterinary faculty and staff members. All participants were required to be at least 18 years of age and reside in the United States.

The questionnaire was developed using standardized survey software (Qualtrics ®) and was adapted from Gruen et al.^1^. The adapted questionnaire was piloted with a small sample of students and veterinarians to provide feedback on question flow and readability. Additionally, a psychometrician (KR) evaluated survey items for readability and ambiguity. Feedback from the pilot survey was incorporated into the final version of the questionnaire.

**Figure 1:**
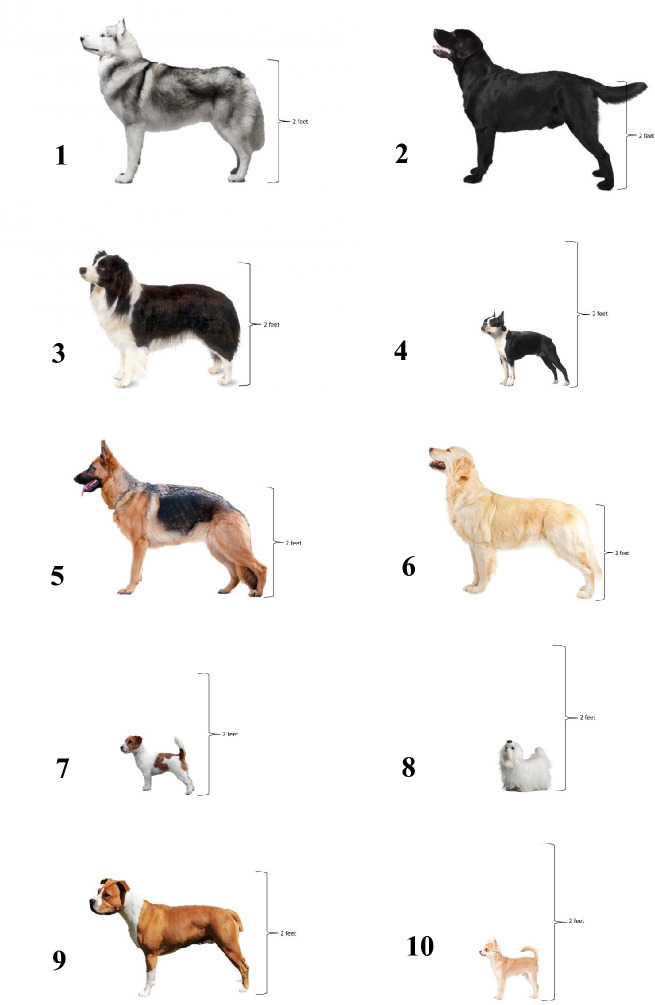
Standardized pictures presented to survey participants of 10 dog breeds: (1) Siberian husky, (2) Labrador retriever, (3) border collie, (4) Boston terrier, (5) German shepherd, (6) golden retriever, (7) Jack Russell terrier, (8) Maltese, (9) pitbull type dog, and (10) Chihuahua. These breeds were selected as veterinarians and the general public rated these dogs differently in terms of their pain sensitivity.

Adapted from Gruen et al.^1^, 10 purebred dog breeds were selected to encompass dogs whose pain sensitivity ratings had previously shown significantly different between the veterinarian and general public populations; belonged to different classifications of pain ratings by veterinarians (e.g., rated as having high sensitivity, average sensitivity, and low sensitivity); and were of varying sizes. The same standardized pictures of dogs from each of these breeds were used in the adapted questionnaire (Fig. 1).

**Figure 2:**
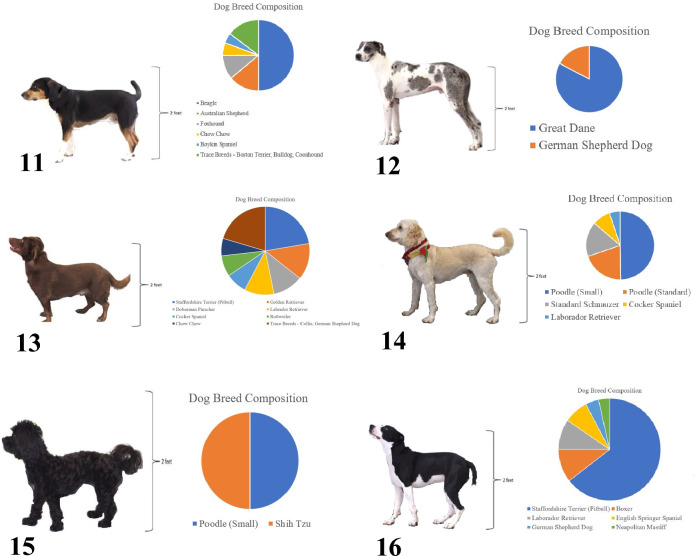
Standardized pictures of six mixed breed dogs with Embark® DNA profiles. Presentation of DNA profiles was randomly assigned to survey participants. Form A presented dogs 11, 12, and 13 with their DNA profile and presented dogs 14, 15, and 16 without their DNA profile. Form B presented dogs 11, 12, and 13 without their DNA profile and presented dogs 14, 15, and 16 with their DNA profile.

Pictures of six mixed breed dogs were standardized (e.g., set to all face the same direction, same background) and included (Fig. 2) in the questionnaire. All mixed breed dogs had breed composition analysed using an Embark® DNA kit. For all dogs, a scale was provided as reference for height and was set according to the dog’s height at withers.

Two forms of the survey were developed to randomize presentation of DNA profiles for the 6 mixed breed dogs (Fig. 2). The survey questionnaire was presented in seven blocks, as follows: (1) informed consent; (2) an explanation of the pain sensitivity rating scale and example; (3) presentation of the 10 purebred dog breeds and 6 mixed breed dogs and pain sensitivity rating scale, with 3 mixed breed dogs randomly presented with their DNA profile and 3 mixed breed dogs randomly presented without their DNA profile; (4) presentation of the 10 purebred dog breeds and 6 mixed breed dogs and trust rating questions, with 3 mixed breed dogs randomly presented with their DNA profile and 3 mixed breed dogs randomly presented without their DNA profile; (5) a feelings thermometer, where respondents were asked to rate how warmly or coolly they felt about the 10 purebred dog breeds and different groups of dogs on a scale of 0 (cool) to 100 (warm) with 50 as neutral; (6) demographic questions; and (7) an opportunity to provide feedback about the survey in general.

**Figure 3:**
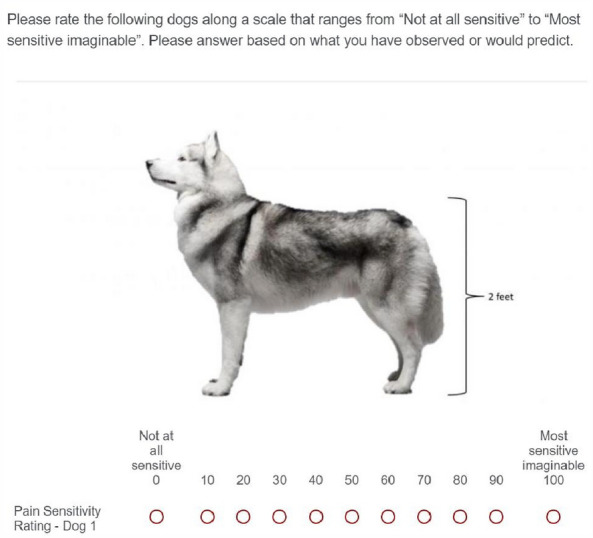
An example question from block 3 in the survey questionnaire. Participants were asked to rate how sensitive to pain they believed each dog to be on an 11-point Likert scale. Methodology adapted from Gruen et al.^1^.

Survey respondents were provided with general directions and asked to rate 10 dog breeds and 6 mixed breed dogs on an 11-point Likert scale using radio buttons from 0 “Not at all sensitive” to 100 “Most sensitive imaginable” (Fig. 3). In the next block, participants were asked to rate how trustworthy they felt that the 10 dog breeds and 6 mixed breed dogs were in five different scenarios (see Supplementary Fig. S1). Participants were requested to rate their trustworthiness in each dog using radio buttons on an 11-point Likert scale from 0 “Not at all likely” to 10 “Very likely”. Finally, participant’s attitudes towards the 10 dog breeds and dog groups were measured using a feelings thermometer^31,32^ (see Supplementary Fig. S2). Participants were asked to use a sliding scale tool to indicate how warmly or coolly they felt towards each dog breed and dog group provided on a scale of 0 (cool) to 100 (warm) with 50 representing neutral feelings.

Demographic information was collected from all participants. General public participants reported their age, race/ethnicity, gender, region of residence in the United States, highest level of education, and annual household income. All students, as well as veterinary school faculty and staff, reported their age, race/ethnicity, and gender. Undergraduate students reported their year in school, major, whether they were planning to pursue a veterinary degree following graduation, and if they had previous experience working at a veterinary clinic. Veterinary students reported their year in veterinary school, their interests in veterinary medicine, and if they had previous experience working at a veterinary clinic. Veterinary faculty and staff reported their specialty and degrees obtained. Any faculty/staff who had reported earning a veterinary degree were asked to indicate how much time has passed since they graduated, and what region in the United States they had obtained their veterinary degree.

### Statistical analysis

Data analysis included descriptive and inferential statistics. Descriptive statistics were calculated for all demographic questions and examined by participant population. Participant populations included general public, undergraduates, 1st and 2nd-year veterinary students, 3rd and 4th-year veterinary students, and veterinary faculty and staff. The decision to group 1st and 2nd-year veterinary students and 3rd and 4th-year veterinary students was made in order to best classify veterinary students based on their course work. Across veterinary schools, the 1st and 2nd-year curriculum focuses largely on didactic material while the 3rd and 4th years engage students in applied or clinical learning.

To answer all questions of interest, linear mixed-effects regression models were used that accounted for “repeated measures” in R software (R Core Team). A nested linear mixed effects regression model was used to assess breed, survey form, and their interaction on pain sensitivity ratings accounting for a random effect for subject and university. An ANOVA using Satterthwaite’s method to calculate degrees of freedom was used to further evaluate the effect of feelings thermometer scores, participant population, and their interaction for pain sensitivity ratings. A linear mixed model was used to assess pain sensitivity ratings as predicted by the interaction between the feelings thermometer scores and participant population. In the model, subject and university were accounted for as random effects. The effects of participant population, breed, and their interaction for pain sensitivity ratings were examined using Satterthwaite’s method. The effects of clinical experience, breed, and their interaction for pain sensitivity ratings were examined using ANOVA with degrees of freedom calculated using Satterthwaite’s method. Only the undergraduate participant population was used to evaluate the clinical experience question, as > 90% of veterinary students indicated having clinical experience. Comparisons were made among participant populations using linear contrasts on the regression coefficients of linear mixed effects models.

P-values are reported as summary statistics and no corrections for multiple testing have been used. P-values should be interpreted with caution as multiple comparisons were not performed. Throughout, we try to avoid making binary decisions about statistical significance. Confidence intervals are used to help interpret the scientific significance of findings.

## Results

### Demographics

The final participant sample included 2210 individuals representing 1020 members of the general public, 361 undergraduates, 308 1st and 2nd-year veterinary students, 228 3rd and 4th-year veterinary students, and 293 veterinary faculty and staff members. The veterinary participant population included veterinary students, faculty, and staff members from seven of the eight veterinary colleges that were administered the survey. Less than 10 responses were received from veterinary students, faculty, and staff from one university; therefore, the decision was made to not include these responses in the final sample.

For the general public, participants were quite diverse in many areas with the exception of highest level of education obtained (see Supplementary Tables S1, S2). The sample reflected general public participants with higher levels of education than expected from the general population of the United States.

The undergraduate sample was purposefully recruited and therefore reflects higher participation from Animal Science, Zoology and Biology majors (Supplementary Table S3). These majors are pursued at a higher rate by female students at North Carolina State University^33–35^, and this is reflected in the sample (see Supplementary Table S1). The majority of undergraduates (84.2%) reported being in their 2nd, 3rd, or 4th year of their degree program. Roughly half of undergraduates were considering pursuing a veterinary degree following graduation with 45.4% of the undergraduate respondents reporting having prior experience working in a veterinary clinic (see Supplementary Table S3).

Veterinary student, faculty and staff participants largely reflected the demographics of the profession. However, females were slightly overrepresented in the present study (see Supplementary Table S1). The majority of veterinary students (~ 93%) had prior clinical experience (see Supplementary Table S4). Further details about the veterinary student and veterinary faculty and staff participant populations can be found in Supplementary Tables S4 and S5.

### Pain sensitivity ratings differ when the DNA profile is known for some mixed-breed dogs

Information about a dog’s DNA profile affected pain sensitivity ratings, χ^2^ (6) = 39.535, *p* < 0.001 (Table 1). When breed composition was shown for the six mixed breed dogs, participants rated Dog 11 (*p* = 0.018) and Dog 15 (*p* = 0.021) as more sensitive to pain and Dog 16 as less sensitive to pain (*p* < 0.001).

**Table 1:** Comparison between pain sensitivity ratings for mixed breeds dogs as presented on survey forms A and B using a linear mixed effects model with fixed effects for form, breed, and their interaction and random effects for individual.

Mixed breed dog	Estimate	Std. error	Z-statistic	*p*-value
Dog 11 (50% Beagle, 13.9% Australian shepherd)	− 1.989	0.841	− 2.361	0.018*
Dog 12 (82.7% Great Dane, 17.3% German shepherd)	− 1.452	0.841	− 1.726	0.084
Dog 13 (22.3% Staffordshire terrier (pitbull), 20.4% trace breeds)	− 1.027	0.841	− 1.221	0.222
Dog 14 (49.8% Poodle—small, 20.0% Poodle—standard)	− 0.850	0.841	− 1.011	0.312
Dog 15 (50% Poodle—small, 50% Shih tzu)	1.941	0.841	2.307	0.021*
Dog 16 (64.5% Staffordshire terrier (pitbull), 10.5% Boxer)	− 3.057	0.841	− 3.634	2.79e−4***

Estimates are obtained through a linear contrast. Estimates represent the estimated average difference between forms B relative to A. Negative estimates indicate that participants with Form B rated dogs as less sensitive to pain compared to participants with Form A. Positive estimates indicate that participants with Form B rated dogs as more sensitive to pain compared to participants with Form A.

*Indicates p ≤ 0.05, ***indicates p ≤ 0.001.

### Pain sensitivity ratings differ by participant population

Participant population had an effect on pain sensitivity ratings, F (4, 2) = 39.429, *p* = 0.025. The general public reported higher pain sensitivity ratings compared to all academic participant populations (*p* < 0.001) (see Supplementary Table S6). Compared to ratings from the general public, academic participant populations had estimated average differences in ratings that were 8.9 to 11.6 points lower. Within the academic participant populations, 3rd and 4th-year veterinary students differed from undergraduate students and veterinary faculty and staff, with 3rd and 4th-year veterinary students reporting lower pain sensitivity ratings (*p* < 0.05; see Supplementary Table S7).

### Feelings thermometer ratings differ by participant population

There was an effect of participant population on feelings thermometer ratings, F (3, 22,151) = 28.750,* p* < 0.001. Compared to the general public, undergraduates did not differ in their feelings thermometer ratings (*p* > 0.05); however, veterinary students and veterinary faculty and staff reported cooler feelings than the general public across dog breeds (*p* < 0.001; see Supplementary Table S8). Compared to ratings from the general public, academic participant populations had estimated average differences in ratings that were 6.1 to 9.8 points lower.

Within the academic participant populations, undergraduate students reported warmer feelings across dog breeds compared to all veterinary participant populations (*p* < 0.001). Veterinary faculty and staff reported cooler feelings than the 1st and 2nd-year veterinary students (*p* = 0.002). However, 3rd and 4th-year veterinary students did not differ in their feelings thermometer ratings compared to veterinary faculty and staff and 1st and 2nd-year students (*p* > 0.05; see Supplementary Table S9).

### Participant population and feelings thermometer ratings influence dog pain sensitivity ratings

**Figure 4:**
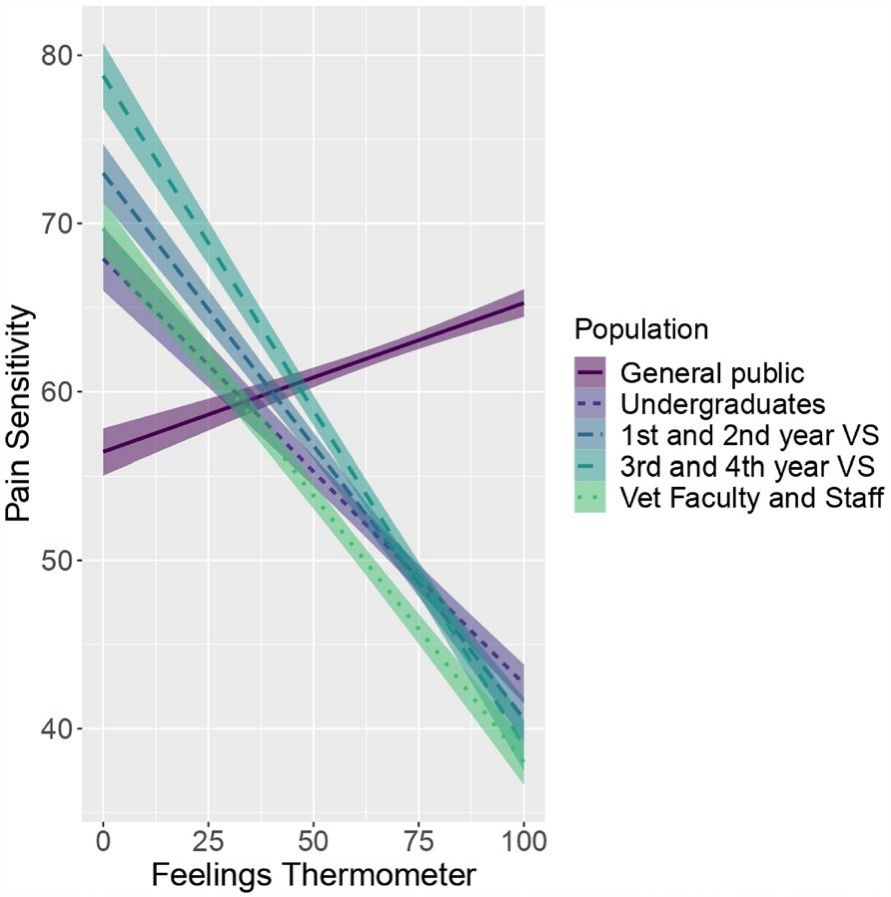
Relationship between dog pain sensitivity ratings and feelings thermometer ratings by participant population. The following abbreviations were used: *VS* student, *vet* veterinary.

Feelings thermometer ratings predicted pain sensitivity ratings (*p* < 0.001). For pain sensitivity ratings, there was an interaction between feelings thermometer ratings and participant population, F (4, 21,818.2) = 36.016, *p* < 0.001 (Fig. 4).

Compared to the general public’s feelings thermometer ratings, all academic participant populations were different (*p* < 0.01) (see Supplementary Table S10). The academic participant populations have negative relationships between pain sensitivity ratings and feelings thermometer ratings (see Supplementary Table S11). As the general public felt warmer towards breeds, ratings of those breeds’ pain sensitivities increased. However, as the academic participant populations felt warmer toward breeds, ratings of those breeds’ pain sensitivities decreased.

### Participant populations differ in their pain sensitivity ratings by breed

**Figure 5:**
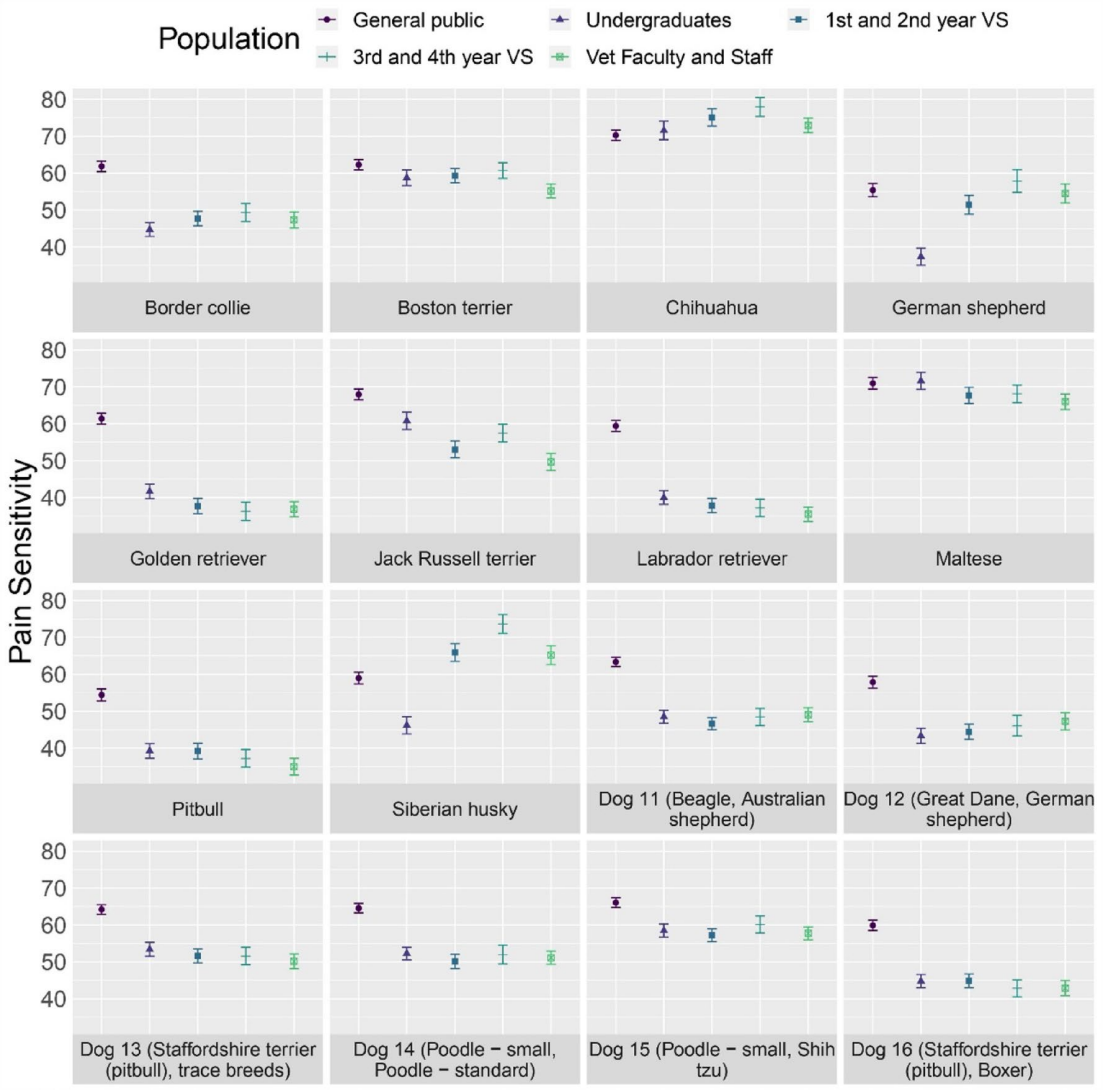
Pain sensitivity ratings (mean with 95% confidence intervals) by participant population for each dog breed. The following abbreviations were used: *VS* student, *vet* veterinary.

Breed also had an effect on pain sensitivity ratings across participant populations, F (15, 33,177) = 496.140, *p* < 0.001). However, the best fit model revealed that there was a breed by participant population interaction, F (60, 33,137) = 42.248, *p* < 0.001 (Fig. 5).

**Figure 6:**
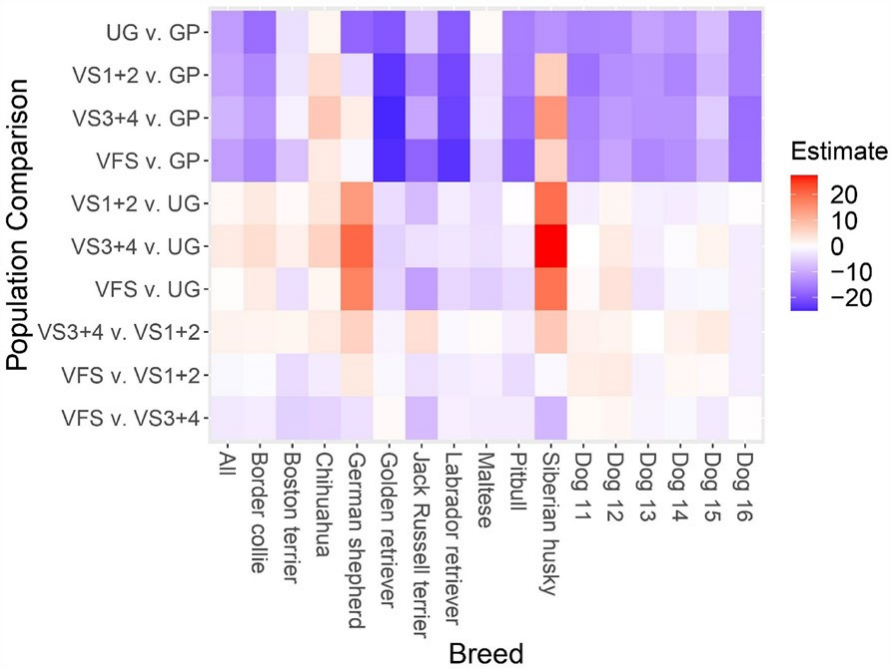
Visualization of the pain sensitivity rating estimates by participant population for each dog breed. Comparisons can be interpreted by the first listed participant population compared to the second listed participant population. Estimate represents the estimated average difference in pain sensitivity rating for the first participant population compared to the second participant population with red indicating the first participant population rated that breed as more sensitive to pain compared to the second participant population and blue indicating the first participant population rated that breed as less sensitive to pain compared to the second participant population. For example, veterinary faculty and staff rated the golden retriever as less sensitive to pain compared to the general public. The following participant population abbreviations were used: *GP* general public, *UG* undergraduates, *VS1* + *2* 1st and 2nd year veterinary students, *VS3* + *4* 3rd and 4th year veterinary students, *VFS* veterinary faculty and staff. Dog 11 = Beagle, Australian shepherd; Dog 12 = Great Dane, German shepherd; Dog 13 = Staffordshire terrier (pitbull), trace breeds; Dog 14 = Poodle (small), poodle (standard); Dog 15 = Poodle (small), shih tzu; Dog 16 = Staffordshire terrier (pitbull), Boxer.

Participant population differences between breeds for pain sensitivity ratings can be further visualized in Fig. 6. For statistical differences, please refer to Supplementary Tables S12 and S13.

**Figure 7:**
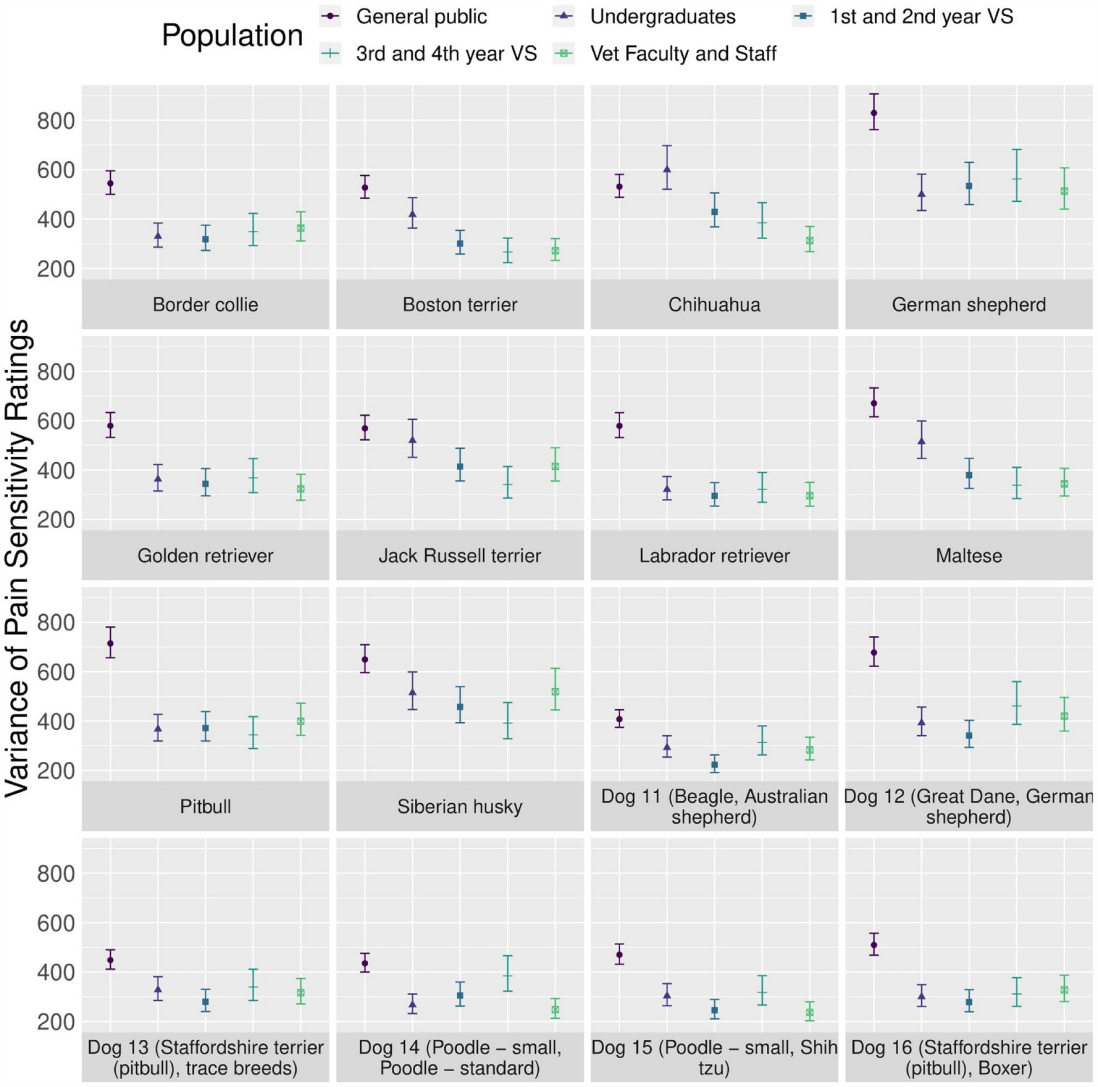
Variance of pain sensitivity ratings (mean with 95% confidence intervals) by participant population for each dog breed. The following abbreviations were used: *VS* student, *vet* veterinary.

Further, variance in pain sensitivity ratings was lower among academic participant populations compared to the general public across all dog breeds (*p* < 0.001). For the majority of dog breeds, the variance was smallest for veterinary faculty and staff, followed by 3rd and 4th year veterinary students, 1st and 2nd year veterinary students, and undergraduates (Fig. 7). Statistical information about the variance in pain sensitivity ratings by participant population is available in Supplementary Tables S14 and S15.

### Clinical experience changes pain sensitivity ratings for some breeds

**Figure 8:**
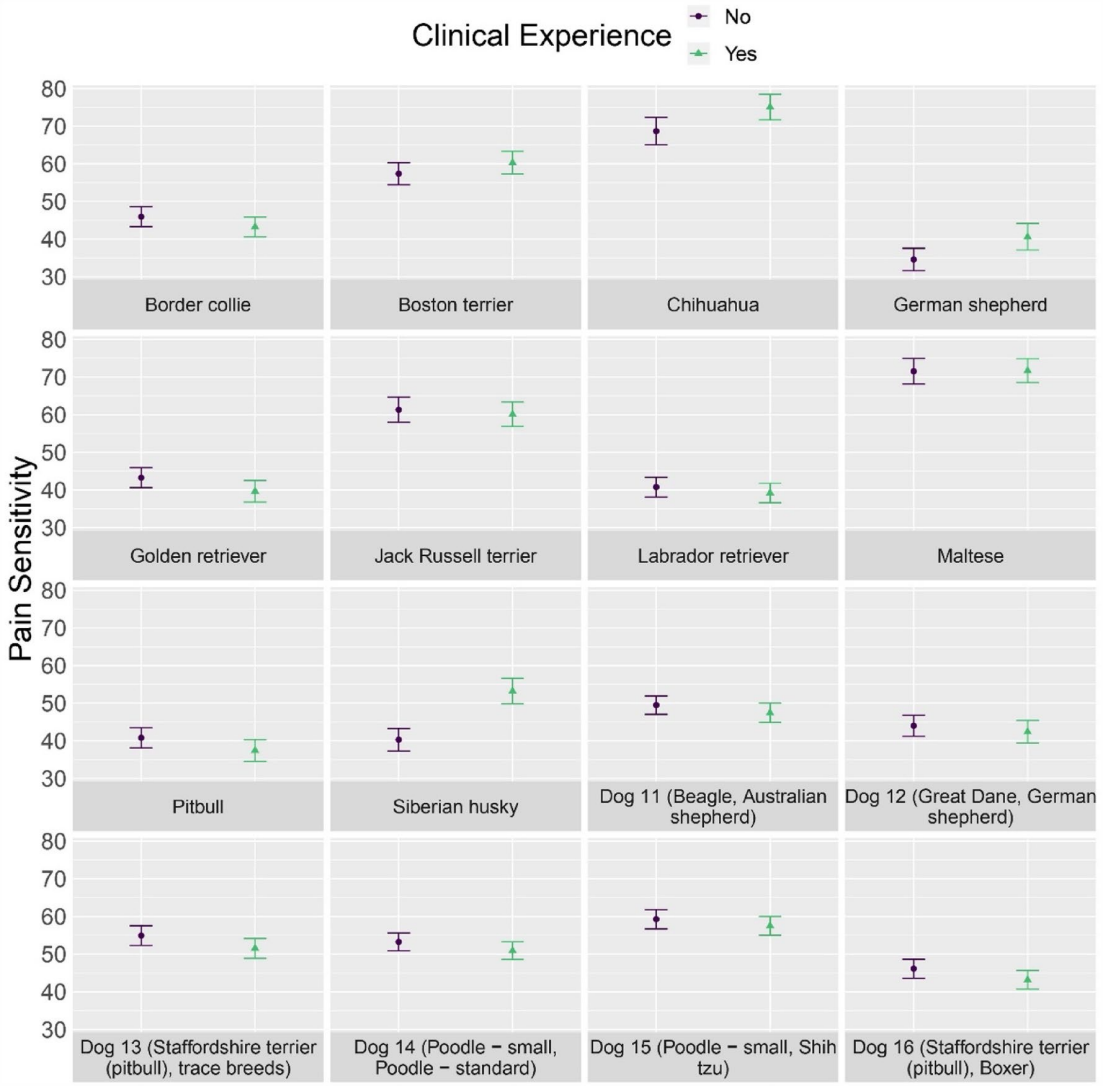
Pain sensitivity ratings (mean with 95% confidence intervals) reported by undergraduates with and without clinical experience for each dog breed.

**Figure 9:**
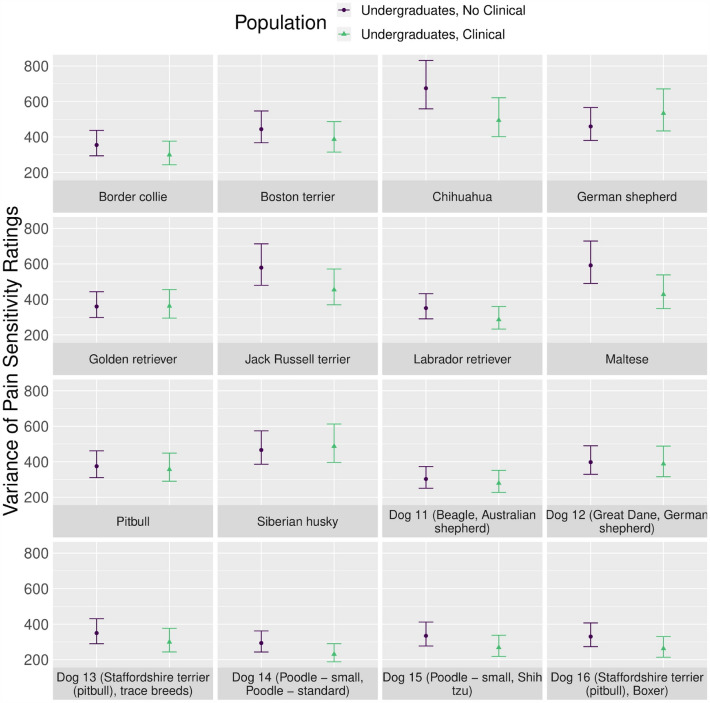
Variance of pain sensitivity ratings (mean with 95% confidence intervals) reported by undergraduates with and without clinical experience for each dog breed.

For pain sensitivity ratings, the interaction between clinical experience and breed was important, F (15, 5385) = 7.061, *p* < 0.001 (Fig. 8). Undergraduates with clinical experience reported higher pain sensitivity scores for Chihuahuas (*p* = 0.012), German shepherds (*p* = 0.011), and Siberian huskies (*p* < 0.001) (see Supplementary Table S16). For undergraduates, clinical experience generally shifted variance to be more aligned with the veterinary students and veterinary faculty and staff (Fig. 9). Differences in the variance of pain sensitivity ratings between undergraduates with and without clinical experience are presented in Supplementary Table S17.

## Discussion

The present study found that veterinary education and experience influence pain sensitivity ratings across dog breeds, thus supporting our predictions. Compared to the general public and undergraduates, veterinary students rate pain sensitivity more similarly to veterinary faculty and staff. The variance for pain sensitivity ratings among the academic participant populations is also smaller compared to the general public with the smallest variance reported by the veterinary faculty and staff, followed by veterinary students and undergraduates. Further, when undergraduates have clinical experience, they begin to rate certain dog breeds (Chihuahuas, German shepherds, Siberian huskies) similar to the veterinary participant populations. These findings suggest that aspects of the veterinary training experience shape individuals’ perceptions about pain across certain dog breeds.

As there was no known physiological explanation for pain sensitivity differences among dog breeds at the time this survey was administered, it is unlikely that veterinary students are being explicitly taught about pain sensitivity related to dog breeds in the formal curriculum, (i.e., planned educational experiences). As our results indicate that students who acquire more education and experience in the field of veterinary medicine report pain sensitivity ratings for dog breeds similar to the veterinary faculty and staff’s ratings, we suggest that students may be learning these dog breed pain sensitivity stereotypes through social processes in the informal or hidden curriculum.

Veterinary colleges are not solely institutions where students learn how to practice medicine. Indeed, similar to other professional schools, they are a critical site of socialization into the field of veterinary medicine^36^. During these 4 years, students are absorbing both intentional and unintentional messages about what it means to be a veterinarian. The unintentional messaging often comes from the hidden curriculum^37,38^. The hidden curriculum encompasses tacit communication from the environment including institutional policies and values (i.e., culture), informal conversation (i.e., slang, humor), and observed behavior (i.e., role modelling)^37,38^. The hidden curriculum is especially powerful in shaping students’ attitudes when they are left unsure about a concept or are presented with conflicting information from their formal education^39–41^. This is when students turn to their experiences and clinical rotations to fill in their gaps in knowledge^40,42^. Prior studies have explored the hidden curriculum of veterinary medicine and identified clinical rotations and clinical service organizations as an integral area responsible for changing student’s professional attitudes, citing interactions with role models and rotation group members as moderators to this relationship^40,41^.

Veterinary faculty and staff’s pain sensitivity ratings across breeds align with prior work which has demonstrated that veterinarians hold breed-specific beliefs about pain sensitivity^1^. Undergraduates who have clinical experience reported pain sensitivity ratings that aligned more closely with the veterinary participant populations for certain dog breeds, suggesting that clinical experience with veterinarians is an important influence on dog breed pain sensitivity stereotype development. Further, as most veterinary students have clinical experience prior to entering veterinary school, it is likely that veterinary students are coming into their veterinary education with dog breed pain sensitivity stereotypes (as evidenced by ratings from 1st and 2nd year veterinary students) and that these stereotypes are becoming solidified through their interactions with interns, residents, and veterinary faculty and staff (i.e., through the hidden curriculum). This suggests that future research should focus on clinical rotations and experiences to ascertain how students are learning these dog-breed pain sensitivity stereotypes.

Additionally, veterinary participant populations reported cooler feelings across dog breeds compared to the general public and undergraduates. Interestingly, 3rd and 4th year veterinary students reported similar feelings to the veterinary faculty and staff. While there are many possible explanations for cooler feelings reported by the veterinary participant populations, these cooler feelings may reflect the experience of working with dogs within the veterinary profession. Students and veterinary faculty and staff learning and working in clinical environments more frequently encounter dogs in a state of discomfort, fear, or pain (i.e., not at their best). In these emotional states, dogs may respond with fearful behaviors including defensive aggression^43,44^ and present challenges for handling, posing a complication to assessment and treatment. It's also possible that veterinary reported feelings thermometer ratings are associated with perceived trust, again deriving from clinical experience.

The relationship between feelings thermometers and pain sensitivity ratings replicates results from Gruen et al.^1^. We once again identified a positive relationship among general public respondents—the more warmly an individual felt toward a breed or breed type, the more sensitive to pain they rated the dog. However, the academic participant populations had a negative relationship indicating that the more warmly an individual felt toward a breed or breed type, the lower they rated their pain sensitivity. This may be reflective of the interpretation of the term “pain sensitivity” in the survey. Individuals with veterinary education or experience may have interpreted pain sensitivity as behavioral reactivity, which can make handling more difficult in a veterinary environment^1^. Veterinarians have previously indicated that they believe dog temperament to greatly influence their sensitivity to pain^1^. Future work should investigate, explicitly, what veterinarians are considering when rating pain sensitivity. Regardless, pain sensitivity beliefs are particularly important to understand in the veterinary participant populations, as they could affect pain recognition and treatment in patients.

For all mixed breed dogs, the general public reported higher pain sensitivity than the academic participant populations; however, the academic participant populations generally reported similar pain sensitivity for these dogs. This suggests that respondents may have had difficulty employing dog breed stereotypes with dogs of unknown or mixed breed ancestry. This finding may not be surprising since dog breed stereotypes are substantially less likely to influence the perception of a dog’s behavior for mixed breed dogs as they do for purebred dogs^45^. Prior research studies have identified that people, including those who work with dogs professionally, experience difficulty identifying dog breed ancestry from physical traits^45–47^.

However, when breed ancestry was presented, this knowledge did affect the pain sensitivity ratings for certain mixed breed dogs. This may be because the breed composition of these mixed breed dogs included breeds or breed types that respondents held strong stereotypes for. For example, when respondents were shown Dog 16’s breed ancestry (primarily Staffordshire terrier (pitbull) at 64.5%), they reported lower pain sensitivity ratings for this dog compared to respondents who were not presented with breed ancestry information. This is a breed/breed type with widely held stereotypes^48–50^ frequently listed on breed restricted legislature^51–53^. Further, both general public and veterinarian responders rate this breed with low pain sensitivity in previous work^1^ and in the current study. The origin of these beliefs regarding pain sensitivity in pitbulls is unknown. A 1987 article on perioperative analgesia remarked that, “individual or breed-specific stoicism, such as that often attributed to the pit bull terrier, is also an interesting area of research”^54^ however this has not been evaluated to our knowledge. A recent article from our group found that pitbulls have fairly average sensitivity thresholds during sensory testing^55^. Notably, there were no differences in pain sensitivity ratings between respondents presented with and without Dog 13’s breed ancestry information. This may be explained by further examination of Dog 13’s breed composition; while Dog 13 is listed as predominantly Staffordshire terrier (pitbull), this breed/breed type only comprises 22.3% of the dog’s genetic makeup with eight additional breeds present, and none reaching 50% or more.

A major strength of this study is that through purposeful recruitment, we were able to gather diverse samples that are largely reflective of the populations of interest. Additionally, pain sensitivity ratings reported by the general public and veterinary faculty and staff in the present study are largely in alignment with those previously found by Gruen et al.^1^ providing confidence in our findings. However, it must be noted that there was an overrepresentation of females in the veterinary sample, and this may not fully capture the views of male veterinarians or veterinary students. Prior work has documented male veterinarians are less likely to recognize and treat pain compared to their female colleagues^56–58^; however, this has not been recently evaluated. Therefore, future work may consider targeting this population to identify their viewpoints. Further, the present study cannot determine the specific influence of veterinary faculty, staff, and residents or interns on veterinary student pain sensitivity beliefs as these individuals were grouped together into the participant population of veterinary faculty and staff. This decision was made to increase power and is considered acceptable as all of these individuals work together on the clinic floor and therefore, all may play a role in clinical education. Additionally, we cannot comment on whether one specialty differs in pain sensitivity beliefs from another due to a lack of sample size for individual specialties. This leaves area for future work to investigate the impact of specific individual roles and specialities that contribute to dog breed pain stereotypes. As the first study investigating the academic population’s beliefs about dog breed pain sensitivity, this study was cross-sectional in design. Future longitudinal studies about dog breed pain sensitivity stereotypes would be necessary to assess changes in perception over time during training. Still, this study presents important information in its identification of changes in beliefs about dog breed pain stereotypes during veterinary training.

## Conclusions

In summary, our findings suggest that veterinary education and clinical experiences influence pain sensitivity ratings across dog breeds. This is the first study to investigate changes in beliefs about dog breed pain sensitivity during veterinary training. These findings elucidate the importance of the clinical environment in the students’ socialization into the veterinary profession, specifically related to pain sensitivity beliefs and attitudes towards dogs across various purebred and mixed breeds. Future research is imperative to identify how these dog breed pain sensitivity stereotypes are communicated in the clinical environment, as well as whether these stereotypes affect recognition and treatment of pain by veterinarians.

### Supplementary Information

Supplementary Information.

## Data Availability

The datasets are available from the corresponding author on reasonable request.
