# Supplementary material for: Veterinary education and experience shape beliefs about dog breeds Part 1: Pain sensitivity
Source: Sci Rep. 2023 Aug 24;13:13846. doi: 10.1038/s41598-023-40671-y (PMC10449809; doi:10.1038/s41598-023-40671-y)
Supplement: Supplementary file 1 — Supplementary Information. [file 41598_2023_40671_MOESM1_ESM.docx]

**Supplementary Materials**

**Supplementary Table S1.** Demographic characteristics (gender, race and/or ethnicity, age) of the sample by population. The following abbreviations were used: VS = student and vet = veterinary.

|  | Participant Population  No. (%) | | | | |
| --- | --- | --- | --- | --- | --- |
| Characteristic | General public | Undergraduates | 1^st^ and 2^nd^ Year VS | 3^rd^ and 4^th^ Year VS | Vet Faculty and Staff |
| *Gender* | | | | | |
| Female | 463 (45.4%) | 302 (83.7%) | 178 (91.8%) | 112 (83.6%) | 239 (81.6%) |
| Male | 556 (54.5%) | 54 (15.0%) | 14 (7.2%) | 22 (16.4%) | 49 (16.7%) |
| Other | 1 (0.1%) | 4 (1.1%) | 1 (0.5%) | 0 (0%) | 2 (0.7%) |
| Prefer not to say | 0 (0%) | 0 (0%) | 1 (0.5%) | 0 (0%) | 3 (1.0%) |
| *Race / Ethnicity* | | | | | |
| Asian | 62 (6.1%) | 16 (4.4%) | 9 (4.6%) | 6 (4.5%) | 5 (1.7%) |
| Black or African American | 101 (9.9%) | 23 (6.4%) | 5 (2.6%) | 3 (2.2%) | 8 (2.7%) |
| Caucasian | 745 (73.0%) | 272 (75.3%) | 154 (79.4%) | 112 (83.6%) | 256 (87.4%) |
| Hispanic, Latino or Spanish origin | 60 (5.9%) | 12 (3.3%) | 15 (7.7%) | 7 (5.2%) | 9 (3.0%) |
| Native American or American Indian | 42 (4.1%) | 1 (0.3%) | 2 (1.0%) | 1 (0.7%) | 1 (0.3%) |
| Pacific Islander | 0 (0%) | 0 (0%) | 0 (0%) | 0 (0%) | 1 (0.3%) |
| From multiple races | 10 (1.0%) | 32 (8.9%) | 8 (4.1%) | 5 (3.7%) | 7 (2.4%) |
| Prefer not to say | 0 (0.0%) | 1 (0.3%) | 1 (0.5%) | 0 (0%) | 6 (2.0%) |
| *Age* | | | | | |
| 18 – 29 years | 225 (22.1%) | 357 (98.9%) | 174 (89.7%) | 122 (91.0%) | 54 (18.4%) |
| 30 – 39 years | 368 (36.1%) | 3 (0.8%) | 18 (9.3%) | 11 (8.2%) | 73 (24.9%) |
| 40 – 49 years | 232 (22.7%) | 0 (0%) | 1 (0.5%) | 1 (0.7%) | 48 (16.4%) |
| 50 – 59 years | 126 (12.4%) | 0 (0%) | 0 (0%) | 0 (0%) | 62 (21.2%) |
| 60 – 69 years | 60 (5.9%) | 0 (0%) | 0 (0%) | 0 (0%) | 35 (11.9%) |
| 70 or older | 9 (0.9%) | 0 (0%) | 0 (0%) | 0 (0%) | 10 (3.4%) |
| Prefer not to say | 0 (0%) | 1 (0.3%) | 1 (0.5%) | 0 (0%) | 11 (3.7%) |

**Supplementary Table S2.** Additional demographic characteristics (geographic region, annual household income, highest level of education) of the general population sample.

| Characteristic | No. (%) |
| --- | --- |
| *Geographic Region* | |
| Midwest | 191 (18.7%) |
| Northeast | 220 (21.6%) |
| South | 360 (35.3%) |
| West | 249 (24.4%) |
| *Annual Household Income* | |
| ≤ $24,999 | 84 (8.2%) |
| $25,000 - $39,999 | 147 (14.4%) |
| $40,000 - $59,999 | 276 (27.1%) |
| $60,000 - $79,999 | 223 (21.9%) |
| $80,000 - $99,999 | 149 (14.6%) |
| $100,000 - $149,999 | 103 (10.1%) |
| $150,000 - $199,999 | 26 (2.5%) |
| ≥ $200,000 | 12 (1.2%) |
| *Highest Level of Education* |  |
| Some high school, no diploma | 3 (0.3%) |
| High school graduate, diploma, or equivalent | 65 (6.4%) |
| Some college credit, no degree | 97 (9.5%) |
| Trade / technical / vocational training | 24 (2.4%) |
| Associate degree | 82 (8.0%) |
| Bachelor’s degree | 566 (55.5%) |
| Master’s degree | 176 (17.3%) |
| Doctorate or professional degree | 7 (0.7%) |

**Supplementary Table S3.** Characteristics of the undergraduate sample.

| Characteristic | No. (%) |
| --- | --- |
| *Major* | |
| Animal Sciences | 181 (50.1%) |
| Biology | 109 (30.2%) |
| Zoology | 45 (12.5%) |
| Other | 26 (7.2%) |
| *Year in undergraduate degree program* | |
| 1^st^ Year | 47 (13.0%) |
| 2^nd^ Year | 78 (21.6%) |
| 3^rd^ Year | 117 (32.4%) |
| 4^th^ Year | 109 (30.2%) |
| 5^th^ Year | 9 (2.5%) |
| 6^th^ Year or More | 1 (0.3%) |
| *Considering pursuing a veterinary degree post-graduation* |  |
| Yes | 187 (51.8%) |
| No | 174 (48.2%) |
| *Previous experience working in a veterinary clinic* |  |
| Yes | 164 (45.4%) |
| No | 197 (54.6%) |

**Supplementary Table S4.** Characteristics of the veterinary student sample. The abbreviation VS = veterinary student was used.

|  | Veterinary Student Participant Population  No. (%) | |
| --- | --- | --- |
| Characteristic | 1^st^ and 2^nd^ Year VS | 3^rd^ and 4^th^ Year VS |
| *University attending for veterinary school* | | |
| Auburn | 24 (7.8%) | 13 (5.7%) |
| LSU | 32 (10.4%) | 15 (6.6%) |
| NCSU | 88 (28.6%) | 98 (43.0%) |
| Oregon | 43 (14.0%) | 18 (7.9%) |
| Tufts | 37 (12.0%) | 26 (11.4%) |
| UGA | 44 (14.3%) | 21 (9.2%) |
| V-Tech | 40 (13.0%) | 37 (16.2%) |
| *Year in veterinary school* | | |
| 1^st^ Year | 164 (53.2%) | - |
| 2^nd^ Year | 144 (46.8%) | - |
| 3^rd^ Year | - | 118 (51.8%) |
| 4^th^ Year | - | 110 (48.2%) |
| *Areas of veterinary practice interest* | | |
| Small animal | 191 (62.0%) | 149 (65.4%) |
| Mixed animal | 137 (44.5%) | 62 (27.2%) |
| Food animal | 41 (13.3%) | 36 (15.8%) |
| Zoo / Wildlife | 76 (24.7%) | 45 (19.7%) |
| Laboratory animal | 17 (5.5%) | 18 (7.9%) |
| Undecided | 26 (8.4%) | 4 (1.8%) |
| *Previous experience working in a veterinary clinic* | | |
| Yes | 284 (92.2%) | 214 (93.9%) |
| No | 24 (7.8%) | 14 (6.1%) |

**Supplementary Table S5.** Characteristics of the veterinary faculty and staff sample.

| Characteristic | No. (%) |
| --- | --- |
| *University employed by* |  |
| Auburn | 34 (11.6%) |
| LSU | 16 (5.5%) |
| NCSU | 173 (59.0%) |
| Oregon | 16 (5.5%) |
| Tufts | 53 (18.1%) |
| UGA | 1 (0.3%) |
| V-Tech | 0 (0.0%) |
| *Veterinary specialty / area of expertise employed in* |  |
| Administration | 14 (4.8%) |
| Anatomy | 3 (1.0%) |
| Anaesthesia | 16 (5.5%) |
| Animal welfare and behavior | 7 (2.4%) |
| Dermatology | 3 (1.0%) |
| Emergency and critical care | 27 (9.2%) |
| Exotics / wildlife | 5 (1.7%) |
| Food animal / large animal medicine | 9 (3.1%) |
| Hospitalized patient care | 4 (1.4%) |
| Infectious disease | 3 (1.0%) |
| Internal medicine including cardiology, neurology, and oncology | 55 (18.8%) |
| Laboratory animal medicine | 19 (6.5%) |
| Microbiology | 6 (2.0%) |
| Nutrition | 3 (1.0%) |
| Ophthalmology | 4 (1.4%) |
| Pathology | 15 (5.1%) |
| Pharmacology | 3 (1.0%) |
| Preventative medicine | 15 (5.1%) |
| Public health | 4 (1.4%) |
| Radiology | 13 (4.4%) |
| Research | 14 (4.8%) |
| Sports medicine and rehabilitation | 4 (1.4%) |
| Surgery | 15 (5.1%) |
| Theriogenology | 10 (3.4%) |
| Other | 22 (7.5%) |
| *Degrees obtained* |  |
| Associate’s degree | 64 (21.8%) |
| Bachelor’s degree | 186 (63.5%) |
| Master’s degree | 72 (24.6%) |
| Doctor of Philosophy (PhD) | 47 (16.0%) |
| Doctor of Veterinary Medicine (DVM) | 146 (49.8%) |
| *For those who have obtained a DVM, how much time has passed since veterinary school graduation* | |
| Less than 1 year | 12 (8.2%) |
| 1 – 5 years | 33 (22.6%) |
| 6 – 10 years | 16 (11.0%) |
| 11 – 15 years | 16 (11.0%) |
| 16 – 20 years | 10 (6.8%) |
| More than 20 years | 59 (40.4%) |
| *For those who have obtained a DVM, region of the United States veterinary degree was obtained from* | |
| Midwest | 29 (19.9%) |
| Northeast | 26 (17.8%) |
| South | 49 (33.6%) |
| West | 16 (10.9%) |
| Outside of the United States | 26 (17.8%) |

**Supplementary Table S6. Pain sensitivity ratings – General public comparisons.** Participant population group comparisons between the general public and each academic participant populations for dog pain sensitivity ratings using a linear mixed effects model with fixed effects for participant population and random effects for the individual. Estimate represents the estimated average difference in pain sensitivity rating for the academic participant population of interest compared to the general public. Negative estimates indicate that the academic participant populations rated dogs as less sensitive to pain compared to the general public participant population. Positive estimates indicate that the academic participant populations rated dogs as more sensitive to pain compared to the general public participant population.

| Participant Population Comparison | Estimate | Std. Error | Degrees of Freedom | t-statistic | *p*-value |
| --- | --- | --- | --- | --- | --- |
| General public v. Undergraduates | -11.600 | 0.825 | 2210 | -14.100 | 2.71e-43 |
| General public v. 1^st^ and 2^nd^ Year VS | -10.600 | 0.877 | 2210 | -12.000 | 1.97e-32 |
| General public v. 3^rd^ and 4^th^ Year VS | -8.880 | 0.988 | 2210 | -8.980 | 5.46e-19 |
| General public v. Vet Faculty and Staff | -11.400 | 0.887 | 2210 | -12.900 | 1.46e-36 |

**Supplementary Table S7. Pain sensitivity ratings - Academic population comparisons.** Academic participant population comparisons for dog pain sensitivity ratings using a linear mixed effects model with fixed effects for participant population and random effects for the individual. Estimates were obtained using linear contrasts. Estimate represents the estimated average difference in pain sensitivity rating for the participant population with more training compared to the participant population with less training. Negative estimates indicate that the participant population with less training rated dogs as less sensitive to pain compared to the participant population with more training. Positive estimates indicate that the participant population with less training rated dogs as more sensitive to pain compared to the participant population with more training.

| Participant Population Comparison | Estimate | Std. Error | z-statistic | *p*-value |
| --- | --- | --- | --- | --- |
| Undergraduates v. 1^st^ and 2^nd^ Year VS | 1.067 | 1.045 | 1.021 | 0.308 |
| Undergraduates v. 3^rd^ and 4^th^ Year VS | 2.757 | 1.140 | 2.418 | 0.016 |
| Undergraduates v. Vet Faculty and Staff | 0.226 | 1.054 | 0.214 | 0.831 |
| 1^st^ and 2^nd^ Year VS v. 3rd and 4th VS | 1.690 | 1.178 | 1.434 | 0.151 |
| 1^st^ and 2^nd^ Year VS v. Vet Faculty and Staff | -0.841 | 1.095 | -0.768 | 0.442 |
| 3^rd^ and 4^th^ Year VS v. Vet Faculty and Staff | -2.532 | 1.186 | -2.135 | 0.033 |

**Supplementary Table S8. Feelings thermometers – General public comparisons.** Participant population comparisons between the general public and academic participant populations for feelings thermometer ratings using a linear mixed effects model with fixed effects for participant population and random effects for the individual. Estimate represents the estimated average difference in pain sensitivity rating for the academic participant population of interest compared to the general public participant population. Negative estimates indicate that the academic participant population reported cooler feelings across dog breeds compared to the general public participant population. Positive estimates indicate that the academic participant population reported warmer feelings across dog breeds compared to the general public participant population.

| Participant Population Comparison | Estimate | Std. Error | Degrees of Freedom | t-statistic | *p*-value |
| --- | --- | --- | --- | --- | --- |
| General public v. Undergraduates | 0.047 | 0.890 | 2210 | 0.053 | 0.958 |
| General public v. 1^st^ and 2^nd^ Year VS | -6.120 | 0.946 | 2210 | -6.470 | 1.22e-10 |
| General public v. 3^rd^ and 4^th^ Year VS | -7.950 | 1.070 | 2210 | -7.460 | 1.26e-13 |
| General Public v. Vet Faculty and Staff | -9.850 | 0.957 | 2210 | -10.300 | 2.80e-24 |

**Supplementary Table S9. Feelings thermometers – Academic population comparisons.** Academic participant population comparisons for feelings thermometer ratings using a linear mixed effects model with fixed effects for participant population and random effects for the individual. Estimates are obtained using linear contrasts. Estimate represents the estimated average difference in pain sensitivity rating for the participant population with more training compared to the participant population with less training. Negative estimates indicate that the participant population with more training reported cooler feelings across dog breeds compared to the participant population less training. Positive estimates indicate that the participant population with more training reported warmer feelings across dog breeds compared to the participant population with less training.

| Participant Population Comparison | Estimate | Std. Error | z-statistic | *p*-value |
| --- | --- | --- | --- | --- |
| Undergraduates v. 1^st^ and 2^nd^ Year VS | -6.164 | 1.128 | -5.465 | 4.62e-08 |
| Undergraduates v. 3^rd^ and 4^th^ Year VS | -7.994 | 1.230 | -6.499 | 8.10e-11 |
| Undergraduates v. Vet Faculty and Staff | -9.893 | 1.137 | -8.699 | < 2.00e-16 |
| 1^st^ and 2^nd^ Year VS v. 3^rd^ and 4^th^ Year VS | -1.830 | 1.271 | -1.440 | 0.150 |
| 1^st^ and 2^nd^ Year VS v. Vet Faculty and Staff | -3.729 | 1.181 | -3.157 | 0.002 |
| 3^rd^ and 4^th^ Year VS v. Vet Faculty and Staff | -1.899 | 1.279 | -1.484 | 0.138 |

**Supplementary Table S10. Pain sensitivity and feelings thermometers – General public comparisons.** Participant population comparisons between the general public and academic participant populations for the estimated relationship between pain sensitivity ratings and feelings thermometer ratings using a linear mixed effects model with fixed effects for feelings thermometer, participant population, and their interaction, as well as random effects for the individual. Estimate is the difference in slope for the academic participant populations relative to the general public. Negative estimates indicate that the academic participant populations had a more negative relationship between pain sensitivity ratings and feelings thermometer ratings compared to the general public participant population. Positive estimates indicate that the academic participant population had a more positive relationship between pain sensitivity ratings and feelings thermometer ratings compared to the general public participant population.

| Participant Population Comparison | Estimate | Std. Error | Degrees of Freedom | t-statistic | *p*-value |
| --- | --- | --- | --- | --- | --- |
| General public v. Undergraduates | -0.047 | 0.018 | 22100 | -2.590 | 0.009 |
| General public v. 1^st^ and 2^nd^ Year VS | -0.156 | 0.019 | 22000 | -8.270 | 1.42e-16 |
| General public v. 3^rd^ and 4^th^ Year VS | -0.178 | 0.021 | 21900 | -8.480 | 2.40e-17 |
| General Public v. Vet Faculty and Staff | -0.149 | 0.018 | 21900 | -8.400 | 4.85e-17 |

**Supplementary Table S11. Pain sensitivity and feelings thermometers – Academic population comparisons.** Academic participant population comparisons for the estimated relationship between pain sensitivity ratings and feelings thermometer ratings using a linear mixed effects model with fixed effects for feelings thermometer, participant population, and their interaction, as well as random effects for the individual. Estimates are obtained using linear contrasts. Estimate is the difference in slope for the participant population with more training compared to the participant population with less training. Negative estimates indicate that the academic participant population with more training had a more negative relationship between pain sensitivity ratings and feelings thermometer ratings compared to the participant population with less training. Positive estimates indicate that the participant population with more training had a more positive relationship between pain sensitivity ratings and feelings thermometer ratings compared to the participant population with less training.

| Participant Population Comparison | Estimate | Std. Error | z-statistic | *p*-value |
| --- | --- | --- | --- | --- |
| Undergraduates v. 1^st^ and 2^nd^ Year VS | -0.062 | 0.018 | -3.381 | 7.22e-4 |
| Undergraduates v. 3^rd^ and 4^th^ Year VS | -0.122 | 0.020 | -6.191 | 5.97e-10 |
| Undergraduates v. Vet Faculty and Staff | -0.034 | 0.018 | -1.871 | 0.061 |
| 1^st^ and 2^nd^ Year VS v. 3^rd^ and 4^th^ Year VS | -0.060 | 0.020 | -2.983 | 0.003 |
| 1^st^ and 2^nd^ Year VS v. Vet Faculty and Staff | 0.028 | 0.019 | -1.491 | 0.136 |
| 3^rd^ and 4^th^ Year VS v. Vet Faculty and Staff | 0.088 | 0.020 | 4.386 | 1.15e-5 |

**Supplementary Table S12.** Comparisons between the general public and academic participant populations pain sensitivity ratings by dog breed using a linear model. Estimate represents the estimated average difference in pain sensitivity rating for the academic participant population of interest compared to the general public. Negative estimates indicate that the academic participant population rated the dog breed as less sensitive to pain compared to the general public participant population. Positive estimates indicate that the academic participant population rated the dog breed as more sensitive to pain compared to the general public participant population.

| Participant Population Comparison | Estimate | Std. Error | Degrees of Freedom | t-statistic | *p*-value |
| --- | --- | --- | --- | --- | --- |
| *Border collie* | | | | | |
| General public v. Undergraduates | -17.147 | 1.274 | 2212 | -13.460 | 9.40e-40 |
| General public v. 1^st^ and 2^nd^ Year VS | -14.148 | 1.354 | 2212 | -10.451 | 5.49e-25 |
| General public v. 3^rd^ and 4^th^ Year VS | -12.501 | 1.525 | 2212 | -8.195 | 4.18e-16 |
| General public v. Vet Faculty and Staff | -14.519 | 1.369 | 2212 | -10.602 | 1.18e-25 |
| *Boston terrier* | | | | | |
| General public v. Undergraduates | -3.555 | 1.249 | 2212 | -2.846 | 0.004 |
| General public v. 1^st^ and 2^nd^ Year VS | -2.999 | 1.327 | 2212 | -2.259 | 0.024 |
| General public v. 3^rd^ and 4^th^ Year VS | -1.583 | 1.496 | 2212 | -1.058 | 0.290 |
| General public v. Vet Faculty and Staff | -7.100 | 1.343 | 2212 | -2.846 | 1.36e-07 |
| *Chihuahua* | | | | | |
| General public v. Undergraduates | 1.329 | 1.346 | 2212 | 0.988 | 0.323 |
| General public v. 1^st^ and 2^nd^ Year VS | 4.852 | 1.430 | 2212 | 3.393 | 7.03e-4 |
| General public v. 3^rd^ and 4^th^ Year VS | 7.693 | 1.611 | 2212 | 4.775 | 1.92e-6 |
| General public v. Vet Faculty and Staff | 2.698 | 1.446 | 2212 | 1.865 | 0.062 |
| *German shepherd* | | | | | |
| General public v. Undergraduates | -18.054 | 1.577 | 2212 | -11.446 | 1.64e-29 |
| General public v. 1^st^ and 2^nd^ Year VS | -3.973 | 1.676 | 2212 | -2.370 | 0.018 |
| General public v. 3^rd^ and 4^th^ Year VS | 2.449 | 1.889 | 2212 | 1.297 | 0.195 |
| General public v. Vet Faculty and Staff | -0.920 | 1.696 | 2212 | -0.543 | 0.587 |
| *Golden retriever* | | | | | |
| General public v. Undergraduates | -19.725 | 1.305 | 2212 | -15.111 | 3.59e-49 |
| General public v. 1^st^ and 2^nd^ Year VS | -23.688 | 1.387 | 2212 | -17.075 | 1.66e-61 |
| General public v. 3^rd^ and 4^th^ Year VS | -25.110 | 1.563 | 2212 | -16.065 | 5.13e-55 |
| General public v. Vet Faculty and Staff | -24.560 | 1.403 | 2212 | -17.503 | 2.42e-64 |
| Jack Russell terrier | | | | | |
| General public v. Undergraduates | -7.140 | 1.362 | 2212 | -5.244 | 1.72e-7 |
| General public v. 1^st^ and 2^nd^ Year VS | -14.922 | 1.447 | 2212 | -10.313 | 2.18e-24 |
| General public v. 3^rd^ and 4^th^ Year VS | -10.485 | 1.630 | 2212 | -6.431 | 1.54e-10 |
| General public v. Vet Faculty and Staff | -18.276 | 1.464 | 2212 | -12.487 | 1.25e-34 |
| *Labrador retriever* | | | | | |
| General public v. Undergraduates | -19.402 | 1.273 | 2212 | -15.241 | 5.94e-50 |
| General public v. 1^st^ and 2^nd^ Year VS | -21.545 | 1.353 | 2212 | -15.925 | 3.84e-54 |
| General public v. 3^rd^ and 4^th^ Year VS | -22.209 | 1.524 | 2212 | -14.570 | 5.48e-46 |
| General public v. Vet Faculty and Staff | -23.917 | 1.368 | 2212 | -17.478 | 3.55e-64 |
| *Maltese* | | | | | |
| General public v. Undergraduates | 0.689 | 1.404 | 2212 | 0.491 | 0.624 |
| General public v. 1^st^ and 2^nd^ Year VS | -3.246 | 1.492 | 2212 | -2.176 | 0.030 |
| General public v. 3^rd^ and 4^th^ Year VS | -2.827 | 1.681 | 2212 | -1.682 | 0.093 |
| General public v. Vet Faculty and Staff | -4.988 | 1.509 | 2212 | -3.305 | 9.64e-4 |
| *Pitbull* | | | | | |
| General public v. Undergraduates | -15.159 | 1.408 | 2212 | -10.765 | 2.23e-26 |
| General public v. 1^st^ and 2^nd^ Year VS | -15.253 | 1.497 | 2212 | -10.192 | 7.17e-24 |
| General public v. 3^rd^ and 4^th^ Year VS | -17.204 | 1.686 | 2212 | -10.203 | 6.43e-24 |
| General public v. Vet Faculty and Staff | -19.458 | 1.514 | 2212 | -12.854 | 1.59e-36 |
| *Siberian husky* | | | | | |
| General public v. Undergraduates | -12.802 | 1.443 | 2212 | -8.870 | 1.47e-18 |
| General public v. 1^st^ and 2^nd^ Year VS | 6.919 | 1.534 | 2212 | 4.511 | 6.80e-6 |
| General public v. 3^rd^ and 4^th^ Year VS | 14.650 | 1.728 | 2212 | 8.477 | 4.16e-17 |
| General public v. Vet Faculty and Staff | 6.227 | 1.552 | 2212 | 4.014 | 6.18e-5 |
| *Dog 11 (Beagle, Australian shepherd)* | | | | | |
| General public v. Undergraduates | -14.874 | 1.123 | 2212 | -13.248 | 1.32e-38 |
| General public v. 1^st^ and 2^nd^ Year VS | -16.727 | 1.193 | 2212 | -14.019 | 7.73e-43 |
| General public v. 3^rd^ and 4^th^ Year VS | -14.917 | 1.344 | 2212 | -11.096 | 7.02e-28 |
| General public v. Vet Faculty and Staff | -14.319 | 1.207 | 2212 | -11.864 | 1.60e-31 |
| *Dog 12 (Great Dane, German shepherd)* | | | | | |
| General public v. Undergraduates | -14.520 | 1.405 | 2211 | -10.335 | 1.74e-24 |
| General public v. 1^st^ and 2^nd^ Year VS | -13.415 | 1.493 | 2211 | -8.985 | 5.42e-19 |
| General public v. 3^rd^ and 4^th^ Year VS | -11.783 | 1.685 | 2211 | -6.992 | 3.57e-12 |
| General public v. Vet Faculty and Staff | -10.605 | 1.510 | 2211 | -7.022 | 2.89e-12 |
| *Dog 13 (Staffordshire terrier (pitbull), trace breeds)* | | | | | |
| General public v. Undergraduates | -10.808 | 1.187 | 2211 | -9.107 | 1.84e-19 |
| General public v. 1^st^ and 2^nd^ Year VS | -12.577 | 1.263 | 2211 | -9.960 | 6.88e-23 |
| General public v. 3^rd^ and 4^th^ Year VS | -12.627 | 1.421 | 2211 | -8.886 | 1.29e-18 |
| General public v. Vet Faculty and Staff | -14.072 | 1.276 | 2211 | -11.031 | 1.40e-27 |
| *Dog 14 (Poodle – small, Poodle – standard)* | | | | | |
| General public v. Undergraduates | -12.274 | 1.160 | 2212 | -10.585 | 1.41e-25 |
| General public v. 1^st^ and 2^nd^ Year VS | -14.409 | 1.232 | 2212 | -11.693 | 1.09e-30 |
| General public v. 3^rd^ and 4^th^ Year VS | -12.566 | 1.388 | 2212 | -9.050 | 3.05e-19 |
| General public v. Vet Faculty and Staff | -13.402 | 1.246 | 2212 | -10.752 | 2.56e-26 |
| *Dog 15 (Poodle – small, Shih tzu)* | | | | | |
| General public v. Undergraduates | -7.578 | 1.168 | 2211 | -6.488 | 1.07e-10 |
| General public v. 1^st^ and 2^nd^ Year VS | -8.860 | 1.243 | 2211 | -7.129 | 1.37e-12 |
| General public v. 3^rd^ and 4^th^ Year VS | -5.927 | 1.399 | 2211 | -4.238 | 2.35e-5 |
| General public v. Vet Faculty and Staff | -8.333 | 1.256 | 2211 | -6.637 | 4.02e-11 |
| *Dog 16 (Staffordshire terrier (pitbull), Boxer)* | | | | | |
| General public v. Undergraduates | -15.113 | 1.221 | 2212 | -12.377 | 4.55e-34 |
| General public v. 1^st^ and 2^nd^ Year VS | -15.022 | 1.298 | 2212 | -11.576 | 3.95e-30 |
| General public v. 3^rd^ and 4^th^ Year VS | -17.085 | 1.462 | 2212 | -11.685 | 1.18e-30 |
| General public v. Vet Faculty and Staff | -16.982 | 1.313 | 2212 | -12.938 | 5.80e-37 |

**Supplementary Table S13.** Academic participant population comparisons for pain sensitivity ratings by dog breed using a linear model. Estimate represents the estimated average difference in pain sensitivity rating for the participant population with more training compared to the participant population with less training. Negative estimates indicate that the participant population with more training rated the dog breed as less sensitive to pain compared to the participant population with less training. Positive estimates indicate that the participant population with more training rated the dog breed as more sensitive to pain compared to the participant population with less training.

| Participant Population Comparison | Estimate | Std. Error | Degrees of Freedom | t-statistic | *p*-value |
| --- | --- | --- | --- | --- | --- |
| *Border collie* | | | | | |
| Undergraduates v. 1^st^ and 2^nd^ Year VS | 2.999 | 1.614 | 2212 | 1.858 | 0.063 |
| Undergraduates v. 3^rd^ and 4^th^ Year VS | 4.646 | 1.761 | 2212 | 2.639 | 0.008 |
| Undergraduates v. Vet Faculty and Staff | 2.628 | 1.627 | 2212 | 1.615 | 0.106 |
| 1^st^ and 2^nd^ Year VS v. 3^rd^ and 4^th^ Year VS | 1.647 | 1.819 | 2212 | 0.906 | 0.365 |
| 1^st^ and 2^nd^ Year VS v. Faculty and Staff | -0.370 | 1.691 | 2212 | -0.219 | 0.827 |
| 3^rd^ and 4^th^ Year VS v. Faculty and Staff | -2.018 | 1.831 | 2212 | -1.102 | 0.271 |
| *Boston terrier* | | | | | |
| Undergraduates v. 1^st^ and 2^nd^ Year VS | 0.556 | 1.583 | 2212 | 0.352 | 0.725 |
| Undergraduates v. 3^rd^ and 4^th^ Year VS | 1.972 | 1.726 | 2212 | 1.143 | 0.253 |
| Undergraduates v. Vet Faculty and Staff | -3.545 | 1.784 | 2212 | -2.222 | 0.026 |
| 1^st^ and 2^nd^ Year VS v. 3^rd^ and 4^th^ Year VS | 1.416 | 1.784 | 2212 | 0.794 | 0.427 |
| 1^st^ and 2^nd^ Year VS v. Faculty and Staff | -4.102 | 1.658 | 2212 | -2.475 | 0.013 |
| 3^rd^ and 4^th^ Year VS v. Faculty and Staff | -5.518 | 1.795 | 2212 | -3.074 | 0.002 |
| *Chihuahua* | | | | | |
| Undergraduates v. 1^st^ and 2^nd^ Year VS | 3.523 | 1.705 | 2212 | 2.066 | 0.039 |
| Undergraduates v. 3^rd^ and 4^th^ Year VS | 6.364 | 1.860 | 2212 | 3.422 | 6.32e-4 |
| Undergraduates v. Vet Faculty and Staff | 1.369 | 1.719 | 2212 | 0.796 | 0.426 |
| 1^st^ and 2^nd^ Year VS v. 3^rd^ and 4^th^ Year VS | 2.841 | 1.922 | 2212 | 1.479 | 0.139 |
| 1^st^ and 2^nd^ Year VS v. Faculty and Staff | -2.154 | 1.786 | 2212 | -1.206 | 0.228 |
| 3^rd^ and 4^th^ Year VS v. Faculty and Staff | -4.995 | 1.934 | 2212 | -2.583 | 0.010 |
| *German shepherd* | | | | | |
| Undergraduates v. 1^st^ and 2^nd^ Year VS | 14.081 | 1.999 | 2212 | 7.045 | 2.47e-12 |
| Undergraduates v. 3^rd^ and 4^th^ Year VS | 20.503 | 2.180 | 2212 | 9.406 | < 2.00e-16 |
| Undergraduates v. Vet Faculty and Staff | 17.134 | 2.015 | 2212 | 8.504 | < 2.00e-16 |
| 1^st^ and 2^nd^ Year VS v. 3^rd^ and 4^th^ Year VS | 6.422 | 2.253 | 2212 | 2.851 | 0.004 |
| 1^st^ and 2^nd^ Year VS v. Faculty and Staff | 3.053 | 2.093 | 2212 | 1.459 | 0.145 |
| 3^rd^ and 4^th^ Year VS v. Faculty and Staff | -3.369 | 2.267 | 2212 | -1.486 | 0.137 |
| *Golden retriever* | | | | | |
| Undergraduates v. 1^st^ and 2^nd^ Year VS | -3.963 | 1.654 | 2212 | -2.396 | 0.017 |
| Undergraduates v. 3^rd^ and 4^th^ Year VS | -5.386 | 1.804 | 2212 | -2.985 | 0.003 |
| Undergraduates v. Vet Faculty and Staff | -4.835 | 1.667 | 2212 | -2.900 | 0.004 |
| 1^st^ and 2^nd^ Year VS v. 3^rd^ and 4^th^ Year VS | -1.423 | 1.864 | 2212 | -0.763 | 0.445 |
| 1^st^ and 2^nd^ Year VS v. Faculty and Staff | -0.872 | 1.732 | 2212 | -0.503 | 0.615 |
| 3^rd^ and 4^th^ Year VS v. Faculty and Staff | 0.551 | 1.876 | 2212 | 0.294 | 0.769 |
| Jack Russell terrier | | | | | |
| Undergraduates v. 1^st^ and 2^nd^ Year VS | -7.782 | 1.725 | 2212 | -4.511 | 6.80e-6 |
| Undergraduates v. 3^rd^ and 4^th^ Year VS | -3.345 | 1.884 | 2212 | -1.778 | 0.076 |
| Undergraduates v. Vet Faculty and Staff | -11.136 | 1.739 | 2212 | -6.403 | 1.86e-10 |
| 1^st^ and 2^nd^ Year VS v. 3^rd^ and 4^th^ Year VS | 4.437 | 1.944 | 2212 | 2.282 | 0.023 |
| 1^st^ and 2^nd^ Year VS v. Faculty and Staff | -3.354 | 1.807 | 2212 | -1.856 | 0.064 |
| 3^rd^ and 4^th^ Year VS v. Faculty and Staff | -7.791 | 1.957 | 2212 | -3.982 | 7.07e-5 |
| *Labrador retriever* | | | | | |
| Undergraduates v. 1^st^ and 2^nd^ Year VS | -2.143 | 1.613 | 2212 | -1.328 | 0.184 |
| Undergraduates v. 3^rd^ and 4^th^ Year VS | -2.807 | 1.759 | 2212 | -1.596 | 0.111 |
| Undergraduates v. Vet Faculty and Staff | -4.515 | 1.626 | 2212 | -2.777 | 0.006 |
| 1^st^ and 2^nd^ Year VS v. 3^rd^ and 4^th^ Year VS | -0.664 | 1.818 | 2212 | -0.365 | 0.715 |
| 1^st^ and 2^nd^ Year VS v. Faculty and Staff | -2.372 | 1.689 | 2212 | -1.404 | 0.160 |
| 3^rd^ and 4^th^ Year VS v. Faculty and Staff | -1.708 | 1.829 | 2212 | -0.934 | 0.351 |
| *Maltese* | | | | | |
| Undergraduates v. 1^st^ and 2^nd^ Year VS | -3.935 | 1.779 | 2212 | -2.212 | 0.027 |
| Undergraduates v. 3^rd^ and 4^th^ Year VS | -3.516 | 1.940 | 2212 | -1.812 | 0.070 |
| Undergraduates v. Vet Faculty and Staff | -5.677 | 1.793 | 2212 | -3.166 | 0.002 |
| 1^st^ and 2^nd^ Year VS v. 3^rd^ and 4^th^ Year VS | 0.419 | 2.005 | 2212 | 0.209 | 0.834 |
| 1^st^ and 2^nd^ Year VS v. Faculty and Staff | -1.742 | 1.863 | 2212 | -0.935 | 0.350 |
| 3^rd^ and 4^th^ Year VS v. Faculty and Staff | -2.161 | 2.018 | 2212 | -1.071 | 0.284 |
| *Pitbull* | | | | | |
| Undergraduates v. 1^st^ and 2^nd^ Year VS | -0.093 | 1.784 | 2212 | -0.052 | 0.958 |
| Undergraduates v. 3^rd^ and 4^th^ Year VS | -2.045 | 1.946 | 2212 | -1.051 | 0.293 |
| Undergraduates v. Vet Faculty and Staff | -4.298 | 1.799 | 2212 | -2.390 | 0.017 |
| 1^st^ and 2^nd^ Year VS v. 3^rd^ and 4^th^ Year VS | -1.951 | 2.011 | 2212 | -0.970 | 0.332 |
| 1^st^ and 2^nd^ Year VS v. Faculty and Staff | -4.205 | 1.869 | 2212 | -2.250 | 0.025 |
| 3^rd^ and 4^th^ Year VS v. Faculty and Staff | -2.254 | 2.024 | 2212 | -1.114 | 0.266 |
| *Siberian husky* | | | | | |
| Undergraduates v. 1st and 2nd Year VS | 19.721 | 1.829 | 2212 | 10.783 | < 2.00e-16 |
| Undergraduates v. 3rd and 4th Year VS | 27.453 | 1.995 | 2212 | 13.763 | < 2.00e-16 |
| Undergraduates v. Vet Faculty and Staff | 19.030 | 1.844 | 2212 | 10.322 | < 2.00e-16 |
| 1st and 2nd Year VS v. 3rd and 4th Year VS | 7.731 | 2.061 | 2212 | 3.751 | 1.807e-4 |
| 1^st^ and 2^nd^ Year VS v. Faculty and Staff | -0.692 | 1.915 | 2212 | -0.361 | 0.718 |
| 3^rd^ and 4^th^ Year VS v. Faculty and Staff | -8.423 | 2.074 | 2212 | -4.061 | 5.06e-5 |
| *Dog 11 (Beagle, Australian shepherd)* | | | | | |
| Undergraduates v. 1^st^ and 2^nd^ Year VS | -1.852 | 1.423 | 2212 | -1.302 | 0.193 |
| Undergraduates v. 3^rd^ and 4^th^ Year VS | -0.043 | 1.552 | 2212 | -0.028 | 0.978 |
| Undergraduates v. Vet Faculty and Staff | 0.555 | 1.434 | 2212 | 0.387 | 0.699 |
| 1^st^ and 2^nd^ Year VS v. 3^rd^ and 4^th^ Year VS | 1.809 | 1.603 | 2212 | 1.128 | 0.259 |
| 1^st^ and 2^nd^ Year VS v. Faculty and Staff | 2.408 | 1.490 | 2212 | 1.616 | 0.106 |
| 3^rd^ and 4^th^ Year VS v. Faculty and Staff | 0.599 | 1.614 | 2212 | 0.371 | 0.711 |
| *Dog 12 (Great Dane, German shepherd)* | | | | | |
| Undergraduates v. 1^st^ and 2^nd^ Year VS | 1.106 | 1.780 | 2211 | 0.621 | 0.535 |
| Undergraduates v. 3^rd^ and 4^th^ Year VS | 2.737 | 1.944 | 2211 | 1.408 | 0.159 |
| Undergraduates v. Vet Faculty and Staff | 3.915 | 1.795 | 2211 | 2.182 | 0.029 |
| 1^st^ and 2^nd^ Year VS v. 3^rd^ and 4^th^ Year VS | 1.631 | 2.009 | 2211 | 0.812 | 0.417 |
| 1^st^ and 2^nd^ Year VS v. Faculty and Staff | 2.809 | 1.864 | 2211 | 1.507 | 0.132 |
| 3^rd^ and 4^th^ Year VS v. Faculty and Staff | 1.178 | 2.002 | 2211 | 0.583 | 0.560 |
| *Dog 13 (Staffordshire terrier (pitbull), trace breeds)* | | | | | |
| Undergraduates v. 1^st^ and 2^nd^ Year VS | -1.769 | 1.505 | 2211 | -1.175 | 0.240 |
| Undergraduates v. 3^rd^ and 4^th^ Year VS | -1.819 | 1.640 | 2211 | -1.109 | 0.268 |
| Undergraduates v. Vet Faculty and Staff | -3.264 | 1.516 | 2211 | -2.153 | 0.031 |
| 1^st^ and 2^nd^ Year VS v. 3^rd^ and 4^th^ Year VS | -0.050 | 1.696 | 2211 | -0.029 | 0.977 |
| 1^st^ and 2^nd^ Year VS v. Faculty and Staff | -1.495 | 1.576 | 2211 | -0.948 | 0.343 |
| 3^rd^ and 4^th^ Year VS v. Faculty and Staff | -1.445 | 1.706 | 2211 | -0.847 | 0.397 |
| *Dog 14 (Poodle – small, Poodle – standard)* | | | | | |
| Undergraduates v. 1^st^ and 2^nd^ Year VS | -2.135 | 1.469 | 2212 | -1.453 | 0.146 |
| Undergraduates v. 3^rd^ and 4^th^ Year VS | -0.292 | 1.603 | 2212 | -0.182 | 0.856 |
| Undergraduates v. Vet Faculty and Staff | -1.128 | 1.481 | 2212 | -0.762 | 0.446 |
| 1^st^ and 2^nd^ Year VS v. 3^rd^ and 4^th^ Year VS | 1.844 | 1.656 | 2212 | 1.113 | 0.266 |
| 1^st^ and 2^nd^ Year VS v. Faculty and Staff | 1.007 | 1.539 | 2212 | 0.655 | 0.513 |
| 3^rd^ and 4^th^ Year VS v. Faculty and Staff | -0.837 | 1.666 | 2212 | -0.502 | 0.616 |
| *Dog 15 (Poodle – small, Shih tzu)* | | | | | |
| Undergraduates v. 1^st^ and 2^nd^ Year VS | -1.282 | 1.481 | 2211 | -0.865 | 0.387 |
| Undergraduates v. 3^rd^ and 4^th^ Year VS | 1.651 | 1.614 | 2211 | 1.023 | 0.307 |
| Undergraduates v. Vet Faculty and Staff | -0.755 | 1.492 | 2211 | -0.506 | 0.613 |
| 1^st^ and 2^nd^ Year VS v. 3^rd^ and 4^th^ Year VS | 2.933 | 1.669 | 2211 | 1.757 | 0.079 |
| 1^st^ and 2^nd^ Year VS v. Faculty and Staff | 0.527 | 1.551 | 2211 | 0.340 | 0.734 |
| 3^rd^ and 4^th^ Year VS v. Faculty and Staff | -2.406 | 1.679 | 2211 | -1.433 | 0.152 |
| *Dog 16 (Staffordshire terrier (pitbull), Boxer)* | | | | | |
| Undergraduates v. 1^st^ and 2^nd^ Year VS | 0.091 | 1.547 | 2212 | 0.059 | 0.953 |
| Undergraduates v. 3^rd^ and 4^th^ Year VS | -1.972 | 1.688 | 2212 | -1.169 | 0.243 |
| Undergraduates v. Vet Faculty and Staff | -1.869 | 1.560 | 2212 | -1.198 | 0.231 |
| 1^st^ and 2^nd^ Year VS v. 3^rd^ and 4^th^ Year VS | -2.063 | 1.744 | 2212 | -1.183 | 0.237 |
| 1^st^ and 2^nd^ Year VS v. Faculty and Staff | -1.960 | 1.620 | 2212 | -1.210 | 0.226 |
| 3^rd^ and 4^th^ Year VS v. Faculty and Staff | 0.103 | 1.755 | 2212 | 0.059 | 0.953 |

**Supplementary Table S14.** Comparisons between the general public and academic participant populations for the variance of pain sensitivity ratings by dog breed using a variance test. F-statistic represents a ratio of the sample variances.

| Participant Population Comparison | Degrees of Freedom – 1 | Degrees of Freedom – 2 | F-statistic | p-value |
| --- | --- | --- | --- | --- |
| *Border collie* | | | | |
| General public v. Undergraduates | 1217 | 163 | 1.830 | 2.44e-06 |
| General public v. 1^st^ and 2^nd^ Year VS | 1217 | 307 | 1.722 | 1.44e-08 |
| General public v. 3^rd^ and 4^th^ Year VS | 1217 | 227 | 1.570 | 3.16e-05 |
| General public v. Vet Faculty and Staff | 1217 | 298 | 1.508 | 1.83e-05 |
| *Boston terrier* | | | | |
| General public v. Undergraduates | 1217 | 163 | 1.334 | 0.020 |
| General public v. 1^st^ and 2^nd^ Year VS | 1217 | 307 | 1.719 | 1.60e-08 |
| General public v. 3^rd^ and 4^th^ Year VS | 1217 | 227 | 1.938 | 2.41e-09 |
| General public v. Vet Faculty and Staff | 1217 | 298 | 1.903 | 5.41e-11 |
| *Chihuahua* | | | | |
| General public v. Undergraduates | 1217 | 163 | 1.122 | 0.353 |
| General public v. 1^st^ and 2^nd^ Year VS | 1217 | 307 | 1.291 | 0.006 |
| General public v. 3^rd^ and 4^th^ Year VS | 1217 | 227 | 1.440 | 6.918e-5 |
| General public v. Vet Faculty and Staff | 1217 | 298 | 1.771 | 4.40e-9 |
| *German shepherd* | | | | |
| General public v. Undergraduates | 1217 | 163 | 1.551 | 4.84e-4 |
| General public v. 1^st^ and 2^nd^ Year VS | 1217 | 307 | 1.549 | 3.93e-6 |
| General public v. 3^rd^ and 4^th^ Year VS | 1217 | 227 | 1.482 | 3.323e-4 |
| General public v. Vet Faculty and Staff | 1217 | 298 | 1.612 | 7.47e-7 |
| *Golden retriever* | | | | |
| General public v. Undergraduates | 1217 | 163 | 1.624 | 1.263e-4 |
| General public v. 1^st^ and 2^nd^ Year VS | 1217 | 307 | 1.707 | 2.34e-8 |
| General public v. 3^rd^ and 4^th^ Year VS | 1217 | 227 | 1.597 | 1.62e-5 |
| General public v. Vet Faculty and Staff | 1217 | 298 | 1.815 | 1.02e-9 |
| *Jack Russell terrier* | | | | |
| General public v. Undergraduates | 1217 | 163 | 1.268 | 0.055 |
| General public v. 1^st^ and 2^nd^ Year VS | 1217 | 307 | 1.390 | 4.526e-4 |
| General public v. 3^rd^ and 4^th^ Year VS | 1217 | 227 | 1.685 | 1.76e-6 |
| General public v. Vet Faculty and Staff | 1217 | 298 | 1.388 | 5.672e-4 |
| *Labrador retriever* | | | | |
| General public v. Undergraduates | 1217 | 163 | 2.062 | 2.64e-8 |
| General public v. 1^st^ and 2^nd^ Year VS | 1217 | 307 | 1.993 | 1.37e-12 |
| General public v. 3^rd^ and 4^th^ Year VS | 1217 | 227 | 1.832 | 3.88e-8 |
| General public v. Vet Faculty and Staff | 1217 | 298 | 1.991 | 2.81e-12 |
| *Maltese* | | | | |
| General public v. Undergraduates | 1217 | 163 | 1.535 | 6.508e-4 |
| General public v. 1^st^ and 2^nd^ Year VS | 1217 | 307 | 1.731 | 1.06e-8 |
| General public v. 3^rd^ and 4^th^ Year VS | 1217 | 227 | 1.939 | 2.37e-9 |
| General public v. Vet Faculty and Staff | 1217 | 298 | 1.907 | 4.70e-11 |
| *Pitbull* | | | | |
| General public v. Undergraduates | 1217 | 163 | 1.918 | 4.40e-7 |
| General public v. 1^st^ and 2^nd^ Year VS | 1217 | 307 | 1.840 | 2.64e-10 |
| General public v. 3^rd^ and 4^th^ Year VS | 1217 | 227 | 1.986 | 6.74e-10 |
| General public v. Vet Faculty and Staff | 1217 | 298 | 1.711 | 3.07e-8 |
| *Siberian husky* | | | | |
| General public v. Undergraduates | 1217 | 163 | 1.367 | 0.018 |
| General public v. 1^st^ and 2^nd^ Year VS | 1217 | 307 | 1.456 | 6.84e-5 |
| General public v. 3^rd^ and 4^th^ Year VS | 1217 | 227 | 1.701 | 1.17e-6 |
| General public v. Vet Faculty and Staff | 1217 | 298 | 1.283 | 0.008 |
| *Dog 11 (Beagle, Australian shepherd)* | | | | |
| General public v. Undergraduates | 1217 | 163 | 1.493 | 0.001 |
| General public v. 1^st^ and 2^nd^ Year VS | 1217 | 307 | 1.871 | 9.32e-11 |
| General public v. 3^rd^ and 4^th^ Year VS | 1217 | 227 | 1.330 | 0.007 |
| General public v. Vet Faculty and Staff | 1217 | 298 | 1.474 | 5.00e-5 |
| *Dog 12 (Great Dane, German shepherd)* | | | | |
| General public v. Undergraduates | 1217 | 163 | 1.695 | 3.32e-5 |
| General public v. 1^st^ and 2^nd^ Year VS | 1217 | 307 | 1.925 | 1.41e-11 |
| General public v. 3^rd^ and 4^th^ Year VS | 1217 | 226 | 1.426 | 9.765e-4 |
| General public v. Vet Faculty and Staff | 1217 | 298 | 1.568 | 2.96e-6 |
| *Dog 13 (Staffordshire terrier (pitbull), trace breeds)* | | | | |
| General public v. Undergraduates | 1217 | 163 | 1.483 | 0.002 |
| General public v. 1^st^ and 2^nd^ Year VS | 1217 | 306 | 1.585 | 1.30e-6 |
| General public v. 3^rd^ and 4^th^ Year VS | 1217 | 227 | 1.307 | 0.012 |
| General public v. Vet Faculty and Staff | 1217 | 298 | 1.403 | 3.775e-4 |
| *Dog 14 (Poodle – small, Poodle – standard)* | | | | |
| General public v. Undergraduates | 1217 | 163 | 1.863 | 1.28e-6 |
| General public v. 1^st^ and 2^nd^ Year VS | 1217 | 307 | 1.408 | 2.707e-4 |
| General public v. 3^rd^ and 4^th^ Year VS | 1217 | 227 | 1.118 | 0.294 |
| General public v. Vet Faculty and Staff | 1217 | 298 | 1.733 | 1.50e-8 |
| *Dog 15 (Poodle – small, Shih tzu)* | | | | |
| General public v. Undergraduates | 1217 | 163 | 1.693 | 3.44e-5 |
| General public v. 1^st^ and 2^nd^ Year VS | 1217 | 306 | 1.851 | 1.93e-10 |
| General public v. 3^rd^ and 4^th^ Year VS | 1217 | 227 | 1.428 | 8.981e-4 |
| General public v. Vet Faculty and Staff | 1217 | 298 | 1.919 | 3.20e-11 |
| *Dog 16 (Staffordshire terrier (pitbull), Boxer)* | | | | |
| General public v. Undergraduates | 1217 | 163 | 1.928 | 3.59e-7 |
| General public v. 1^st^ and 2^nd^ Year VS | 1217 | 307 | 1.816 | 6.00e-10 |
| General public v. 3^rd^ and 4^th^ Year VS | 1217 | 227 | 1.626 | 7.86e-6 |
| General public v. Vet Faculty and Staff | 1217 | 298 | 1.545 | 6.00e-6 |

**Supplementary Table S15.** Academic participant population comparisons for the variance of pain sensitivity ratings by dog breed using a variance test. F-statistic represents a ratio of the sample variances.

| Participant Population Comparison | Degrees of Freedom – 1 | Degrees of Freedom – 2 | F-statistic | p-value |
| --- | --- | --- | --- | --- |
| *Border collie* | | | | |
| Undergraduates v. 1^st^ and 2^nd^ Year VS | 163 | 307 | 0.941 | 0.668 |
| Undergraduates v. 3^rd^ and 4^th^ Year VS | 163 | 227 | 0.858 | 0.298 |
| Undergraduates v. Vet Faculty and Staff | 163 | 298 | 0.824 | 0.169 |
| 1^st^ and 2^nd^ Year VS v. 3^rd^ and 4^th^ Year VS | 307 | 227 | 0.912 | 0.451 |
| 1^st^ and 2^nd^ Year VS v. Faculty and Staff | 307 | 298 | 0.876 | 0.249 |
| 3^rd^ and 4^th^ Year VS v. Faculty and Staff | 227 | 298 | 0.960 | 0.750 |
| *Boston terrier* | | | | |
| Undergraduates v. 1^st^ and 2^nd^ Year VS | 163 | 307 | 1.288 | 0.060 |
| Undergraduates v. 3^rd^ and 4^th^ Year VS | 163 | 227 | 1.452 | 0.009 |
| Undergraduates v. Vet Faculty and Staff | 163 | 298 | 1.426 | 0.009 |
| 1^st^ and 2^nd^ Year VS v. 3^rd^ and 4^th^ Year VS | 307 | 227 | 1.128 | 0.337 |
| 1^st^ and 2^nd^ Year VS v. Faculty and Staff | 307 | 298 | 1.107 | 0.377 |
| 3^rd^ and 4^th^ Year VS v. Faculty and Staff | 227 | 298 | 0.982 | 0.888 |
| *Chihuahua* | | | | |
| Undergraduates v. 1^st^ and 2^nd^ Year VS | 163 | 307 | 1.151 | 0.295 |
| Undergraduates v. 3^rd^ and 4^th^ Year VS | 163 | 227 | 1.284 | 0.083 |
| Undergraduates v. Vet Faculty and Staff | 163 | 298 | 1.579 | 7.088e-4 |
| 1^st^ and 2^nd^ Year VS v. 3^rd^ and 4^th^ Year VS | 307 | 227 | 1.115 | 0.384 |
| 1^st^ and 2^nd^ Year VS v. Faculty and Staff | 307 | 298 | 1.371 | 0.006 |
| 3^rd^ and 4^th^ Year VS v. Faculty and Staff | 227 | 298 | 1.230 | 0.095 |
| *German shepherd* | | | | |
| Undergraduates v. 1^st^ and 2^nd^ Year VS | 163 | 307 | 0.999 | 0.997 |
| Undergraduates v. 3^rd^ and 4^th^ Year VS | 163 | 227 | 0.949 | 0.723 |
| Undergraduates v. Vet Faculty and Staff | 163 | 298 | 1.039 | 0.771 |
| 1^st^ and 2^nd^ Year VS v. 3^rd^ and 4^th^ Year VS | 307 | 227 | 0.950 | 0.673 |
| 1^st^ and 2^nd^ Year VS v. Faculty and Staff | 307 | 298 | 1.040 | 0.732 |
| 3^rd^ and 4^th^ Year VS v. Faculty and Staff | 227 | 298 | 1.095 | 0.461 |
| *Golden retriever* | | | | |
| Undergraduates v. 1^st^ and 2^nd^ Year VS | 163 | 307 | 1.051 | 0.704 |
| Undergraduates v. 3^rd^ and 4^th^ Year VS | 163 | 227 | 0.983 | 0.914 |
| Undergraduates v. Vet Faculty and Staff | 163 | 298 | 1.118 | 0.410 |
| 1^st^ and 2^nd^ Year VS v. 3^rd^ and 4^th^ Year VS | 307 | 227 | 0.935 | 0.585 |
| 1^st^ and 2^nd^ Year VS v. Faculty and Staff | 307 | 298 | 1.063 | 0.596 |
| 3^rd^ and 4^th^ Year VS v. Faculty and Staff | 227 | 298 | 1.137 | 0.301 |
| *Jack Russell terrier* | | | | |
| Undergraduates v. 1^st^ and 2^nd^ Year VS | 163 | 307 | 1.097 | 0.491 |
| Undergraduates v. 3^rd^ and 4^th^ Year VS | 163 | 227 | 1.329 | 0.048 |
| Undergraduates v. Vet Faculty and Staff | 163 | 298 | 1.095 | 0.501 |
| 1^st^ and 2^nd^ Year VS v. 3^rd^ and 4^th^ Year VS | 307 | 227 | 1.212 | 0.124 |
| 1^st^ and 2^nd^ Year VS v. Faculty and Staff | 307 | 298 | 0.998 | 0.998 |
| 3^rd^ and 4^th^ Year VS v. Faculty and Staff | 227 | 298 | 0.824 | 0.123 |
| *Labrador retriever* | | | | |
| Undergraduates v. 1^st^ and 2^nd^ Year VS | 163 | 307 | 0.967 | 0.816 |
| Undergraduates v. 3^rd^ and 4^th^ Year VS | 163 | 227 | 0.889 | 0.424 |
| Undergraduates v. Vet Faculty and Staff | 163 | 298 | 0.966 | 0.812 |
| 1^st^ and 2^nd^ Year VS v. 3^rd^ and 4^th^ Year VS | 307 | 227 | 0.920 | 0.494 |
| 1^st^ and 2^nd^ Year VS v. Faculty and Staff | 307 | 298 | 0.999 | 0.993 |
| 3^rd^ and 4^th^ Year VS v. Faculty and Staff | 227 | 298 | 1.087 | 0.501 |
| *Maltese* | | | | |
| Undergraduates v. 1^st^ and 2^nd^ Year VS | 163 | 307 | 1.128 | 0.371 |
| Undergraduates v. 3^rd^ and 4^th^ Year VS | 163 | 227 | 1.263 | 0.105 |
| Undergraduates v. Vet Faculty and Staff | 163 | 298 | 1.242 | 0.109 |
| 1^st^ and 2^nd^ Year VS v. 3^rd^ and 4^th^ Year VS | 307 | 227 | 1.120 | 0.365 |
| 1^st^ and 2^nd^ Year VS v. Faculty and Staff | 307 | 298 | 1.102 | 0.400 |
| 3^rd^ and 4^th^ Year VS v. Faculty and Staff | 227 | 298 | 0.984 | 0.900 |
| *Pitbull* | | | | |
| Undergraduates v. 1^st^ and 2^nd^ Year VS | 163 | 307 | 0.959 | 0.774 |
| Undergraduates v. 3^rd^ and 4^th^ Year VS | 163 | 227 | 1.036 | 0.803 |
| Undergraduates v. Vet Faculty and Staff | 163 | 298 | 0.892 | 0.420 |
| 1^st^ and 2^nd^ Year VS v. 3^rd^ and 4^th^ Year VS | 307 | 227 | 1.079 | 0.542 |
| 1^st^ and 2^nd^ Year VS v. Faculty and Staff | 307 | 298 | 0.930 | 0.529 |
| 3^rd^ and 4^th^ Year VS v. Faculty and Staff | 227 | 298 | 0.862 | 0.237 |
| *Siberian husky* | | | | |
| Undergraduates v. 1^st^ and 2^nd^ Year VS | 163 | 307 | 1.065 | 0.637 |
| Undergraduates v. 3^rd^ and 4^th^ Year VS | 163 | 227 | 1.244 | 0.129 |
| Undergraduates v. Vet Faculty and Staff | 163 | 298 | 0.938 | 0.656 |
| 1^st^ and 2^nd^ Year VS v. 3^rd^ and 4^th^ Year VS | 307 | 227 | 1.168 | 0.213 |
| 1^st^ and 2^nd^ Year VS v. Faculty and Staff | 307 | 298 | 0.881 | 0.273 |
| 3^rd^ and 4^th^ Year VS v. Faculty and Staff | 227 | 298 | 0.754 | 0.025 |
| *Dog 11 (Beagle, Australian shepherd)* | | | | |
| Undergraduates v. 1^st^ and 2^nd^ Year VS | 163 | 307 | 1.253 | 0.094 |
| Undergraduates v. 3^rd^ and 4^th^ Year VS | 163 | 227 | 0.891 | 0.433 |
| Undergraduates v. Vet Faculty and Staff | 163 | 298 | 0.987 | 0.934 |
| 1^st^ and 2^nd^ Year VS v. 3^rd^ and 4^th^ Year VS | 307 | 227 | 0.711 | 0.006 |
| 1^st^ and 2^nd^ Year VS v. Faculty and Staff | 307 | 298 | 0.788 | 0.038 |
| 3^rd^ and 4^th^ Year VS v. Faculty and Staff | 227 | 298 | 1.108 | 0.408 |
| *Dog 12 (Great Dane, German shepherd)* | | | | |
| Undergraduates v. 1^st^ and 2^nd^ Year VS | 163 | 307 | 1.136 | 0.342 |
| Undergraduates v. 3^rd^ and 4^th^ Year VS | 163 | 226 | 0.841 | 0.240 |
| Undergraduates v. Vet Faculty and Staff | 163 | 298 | 0.925 | 0.583 |
| 1^st^ and 2^nd^ Year VS v. 3^rd^ and 4^th^ Year VS | 307 | 227 | 0.740 | 0.015 |
| 1^st^ and 2^nd^ Year VS v. Faculty and Staff | 307 | 298 | 0.814 | 0.074 |
| 3^rd^ and 4^th^ Year VS v. Faculty and Staff | 227 | 298 | 1.100 | 0.442 |
| *Dog 13 (Staffordshire terrier (pitbull), trace breeds)* | | | | |
| Undergraduates v. 1^st^ and 2^nd^ Year VS | 163 | 306 | 1.069 | 0.618 |
| Undergraduates v. 3^rd^ and 4^th^ Year VS | 163 | 227 | 0.881 | 0.389 |
| Undergraduates v. Vet Faculty and Staff | 163 | 298 | 0.946 | 0.696 |
| 1^st^ and 2^nd^ Year VS v. 3^rd^ and 4^th^ Year VS | 307 | 227 | 0.824 | 0.116 |
| 1^st^ and 2^nd^ Year VS v. Faculty and Staff | 307 | 298 | 0.885 | 0.289 |
| 3^rd^ and 4^th^ Year VS v. Faculty and Staff | 227 | 298 | 1.074 | 0.565 |
| *Dog 14 (Poodle – small, Poodle – standard)* | | | | |
| Undergraduates v. 1^st^ and 2^nd^ Year VS | 163 | 307 | 0.756 | 0.046 |
| Undergraduates v. 3^rd^ and 4^th^ Year VS | 163 | 227 | 0.600 | 5.774e-4 |
| Undergraduates v. Vet Faculty and Staff | 163 | 298 | 0.930 | 0.611 |
| 1^st^ and 2^nd^ Year VS v. 3^rd^ and 4^th^ Year VS | 307 | 227 | 0.794 | 0.060 |
| 1^st^ and 2^nd^ Year VS v. Faculty and Staff | 307 | 298 | 1.231 | 0.072 |
| 3^rd^ and 4^th^ Year VS v. Faculty and Staff | 227 | 298 | 1.551 | 3.906e-4 |
| *Dog 15 (Poodle – small, Shih tzu)* | | | | |
| Undergraduates v. 1^st^ and 2^nd^ Year VS | 163 | 306 | 1.093 | 0.505 |
| Undergraduates v. 3^rd^ and 4^th^ Year VS | 163 | 227 | 0.844 | 0.248 |
| Undergraduates v. Vet Faculty and Staff | 163 | 298 | 1.133 | 0.354 |
| 1^st^ and 2^nd^ Year VS v. 3^rd^ and 4^th^ Year VS | 307 | 227 | 0.772 | 0.035 |
| 1^st^ and 2^nd^ Year VS v. Faculty and Staff | 307 | 298 | 1.037 | 0.756 |
| 3^rd^ and 4^th^ Year VS v. Faculty and Staff | 227 | 298 | 1.343 | 0.017 |
| *Dog 16 (Staffordshire terrier (pitbull), Boxer)* | | | | |
| Undergraduates v. 1^st^ and 2^nd^ Year VS | 163 | 307 | 0.942 | 0.673 |
| Undergraduates v. 3^rd^ and 4^th^ Year VS | 163 | 227 | 0.843 | 0.247 |
| Undergraduates v. Vet Faculty and Staff | 163 | 298 | 0.801 | 0.115 |
| 1^st^ and 2^nd^ Year VS v. 3^rd^ and 4^th^ Year VS | 307 | 227 | 0.895 | 0.368 |
| 1^st^ and 2^nd^ Year VS v. Faculty and Staff | 307 | 298 | 0.851 | 0.160 |
| 3^rd^ and 4^th^ Year VS v. Faculty and Staff | 227 | 298 | 0.950 | 0.685 |

**Supplementary Table S16.** Pain sensitivity rating comparisons by dog breed between undergraduates that have clinical experience and those that do not using a t-test with unequal variance. Higher estimates indicate higher pain sensitivity ratings and lower estimates indicate lower pain sensitivity ratings.

| Breed | No Clinical Experience  Estimate | Clinical Experience Estimate | Std. Error | Degrees of Freedom | t-statistic | *p*-value |
| --- | --- | --- | --- | --- | --- | --- |
| Border collie | 45.939 | 43.232 | 1.904 | 355.523 | 1.422 | 0.156 |
| Boston terrier | 57.360 | 60.305 | 2.148 | 354.283 | -1.371 | 0.171 |
| Chihuahua | 68.680 | 75.061 | 2.537 | 358.724 | -2.515 | 0.012 |
| German shepherd | 34.569 | 40.610 | 2.363 | 336.666 | -2.556 | 0.011 |
| Golden retriever | 43.249 | 39.634 | 2.008 | 346.881 | 1.800 | 0.073 |
| Jack Russell terrier | 61.320 | 60.122 | 2.389 | 357.610 | 0.501 | 0.616 |
| Labrador retriever | 40.761 | 39.207 | 1.877 | 356.638 | 0.828 | 0.408 |
| Maltese | 71.523 | 71.707 | 2.369 | 358.831 | -0.078 | 0.938 |
| Pitbull | 40.812 | 37.439 | 2.019 | 350.126 | 1.671 | 0.096 |
| Siberian husky | 40.305 | 53.232 | 2.310 | 344.412 | -5.596 | 4.48e-8 |
| Dog 11 (Beagle, Australian shepherd) | 49.492 | 47.439 | 1.800 | 351.733 | 1.141 | 0.255 |
| Dog 12 (Great Dane, German shepherd) | 44.010 | 42.439 | 2.094 | 348.712 | 0.750 | 0.454 |
| Dog 13 (Staffordshire terrier (pitbull), trace breeds) | 54.924 | 51.524 | 1.897 | 355.036 | 1.792 | 0.074 |
| Dog 14 (Poodle – small, Poodle – standard) | 53.249 | 50.915 | 1.701 | 357.582 | 1.372 | 0.171 |
| Dog 15 (Poodle – small, Shih tzu) | 59.289 | 57.500 | 1.825 | 357.074 | 0.981 | 0.327 |
| Dog 16 (Staffordshire terrier (pitbull), Boxer) | 46.142 | 43.171 | 1.809 | 357.292 | 1.642 | 0.101 |

**Supplementary Table S17.** Comparisons for the variance of pain sensitivity ratings by dog breed between undergraduates that have clinical experience and those that do not using a variance test. F-statistic is a ratio of the empirical variances.

| Breed | Degrees of Freedom –  No clinical experience | Degrees of Freedom – Clinical experience | F-statistic | p-value |
| --- | --- | --- | --- | --- |
| Border collie | 196 | 163 | 1.186 | 0.260 |
| Boston terrier | 196 | 163 | 1.147 | 0.364 |
| Chihuahua | 196 | 163 | 1.366 | 0.039 |
| German shepherd | 196 | 163 | 0.862 | 0.319 |
| Golden retriever | 196 | 163 | 0.994 | 0.966 |
| Jack Russell terrier | 196 | 163 | 1.275 | 0.108 |
| Labrador retriever | 196 | 163 | 1.228 | 0.175 |
| Maltese | 196 | 163 | 1.383 | 0.032 |
| Pitbull | 196 | 163 | 1.051 | 0.743 |
| Siberian husky | 196 | 163 | 0.957 | 0.768 |
| Dog 11 (Beagle, Australian shepherd) | 196 | 163 | 1.084 | 0.594 |
| Dog 12 (Great Dane, German shepherd) | 196 | 163 | 1.025 | 0.873 |
| Dog 13 (Staffordshire terrier (pitbull), trace breeds) | 196 | 163 | 1.170 | 0.300 |
| Dog 14 (Poodle – small, Poodle – standard) | 196 | 163 | 1.273 | 0.110 |
| Dog 15 (Poodle – small, Shih tzu) | 196 | 163 | 1.247 | 0.144 |
| Dog 16 (Staffordshire terrier (pitbull), Boxer) | 196 | 163 | 1.258 | 0.129 |


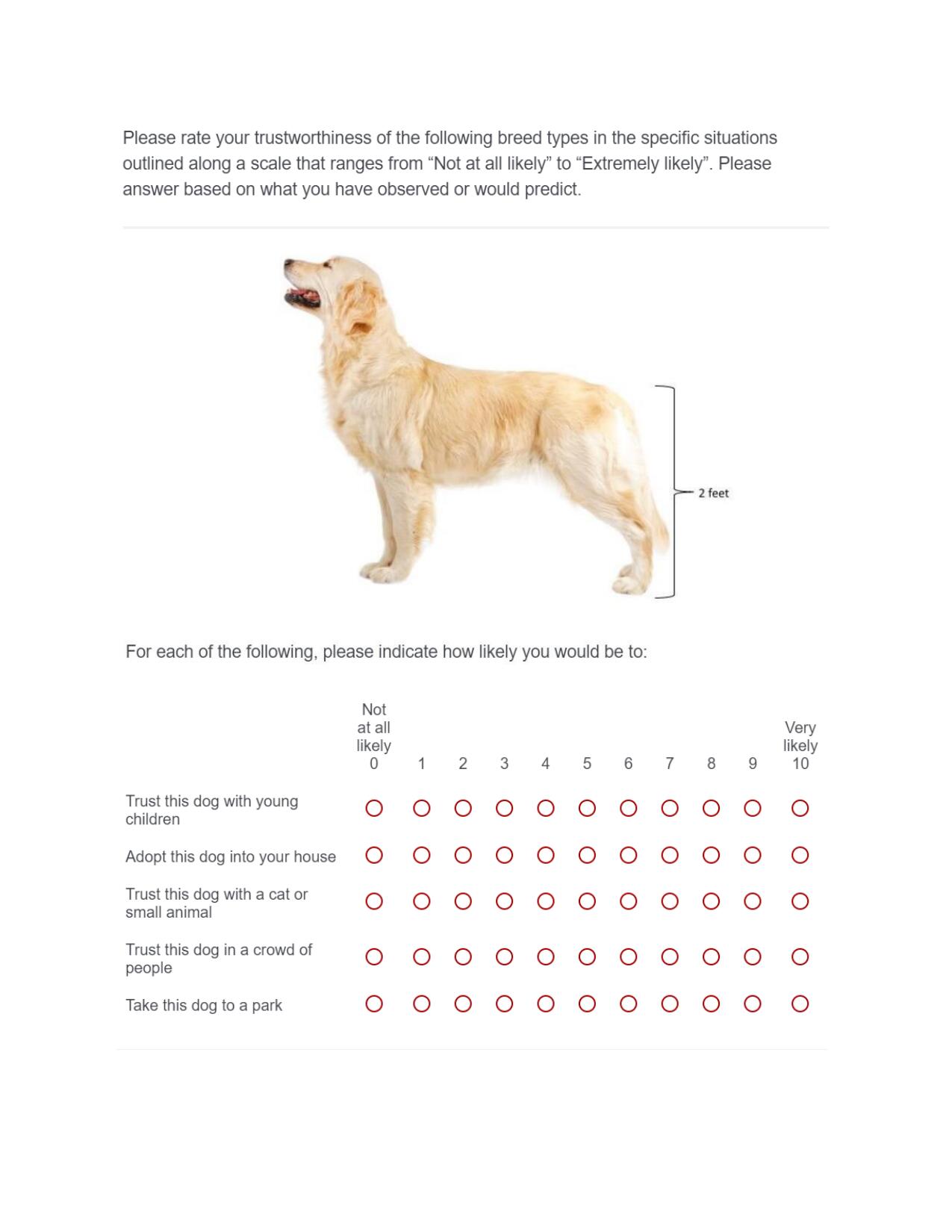


**Supplementary Figure S1.** An example question from block 4 in the survey questionnaire. Participants were asked to rate how trustworthy they believed each dog to be on an 11-point Likert scale within the context of five different scenarios.

**
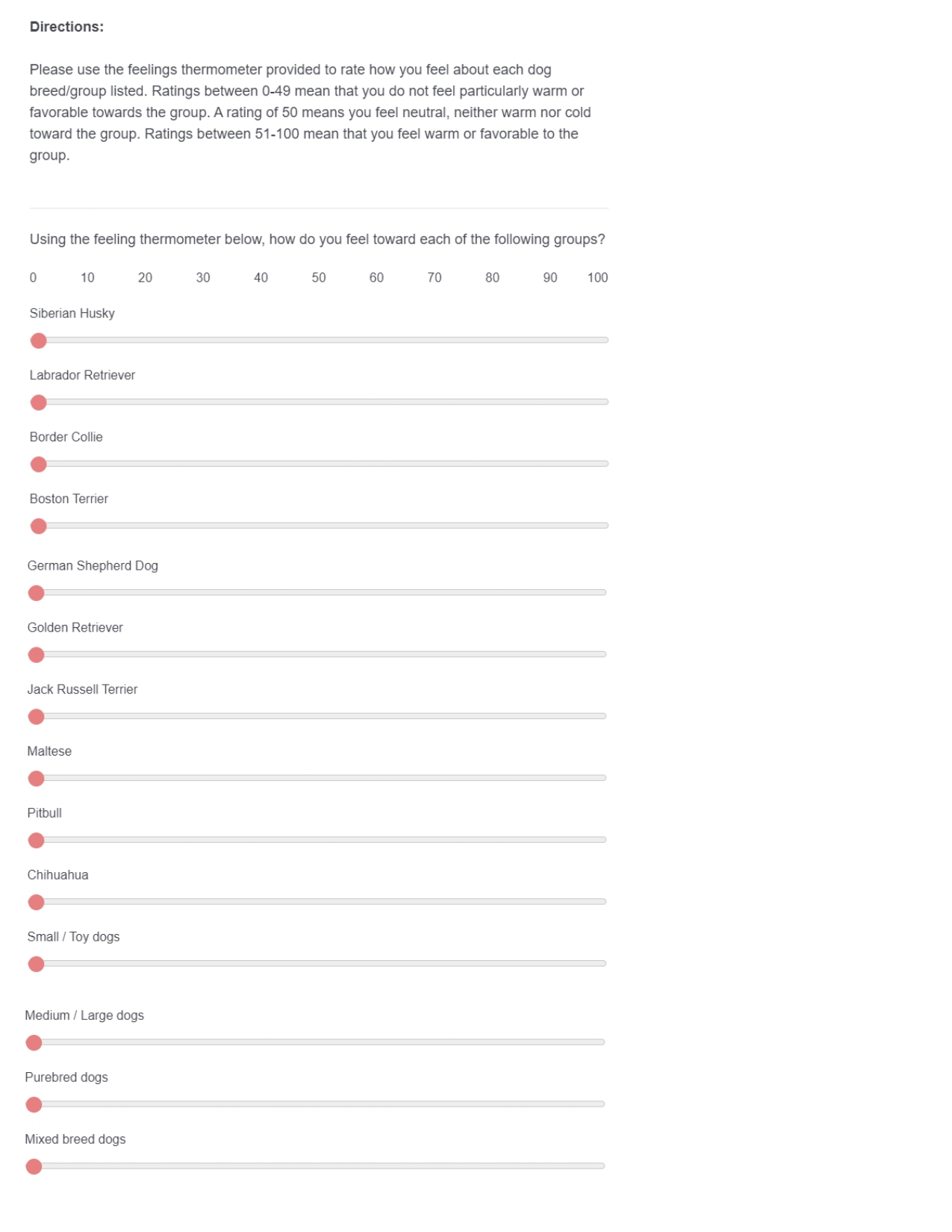
**

**Supplementary Figure S2.** Block 5 from the survey questionnaire in which participants were asked to use a sliding scale tool to rate how warm or cool they felt towards 10 breeds, as well as four groups of dogs.
